# Impact of C-Terminal PKC Phosphorylation on TRPC6 Current Kinetics

**DOI:** 10.3390/ijms262311482

**Published:** 2025-11-27

**Authors:** Maximilian Keck, Sebastian Pöll, Hannah Schmelzer, Tabea Kressmann, Christian Hermann, Michael Mederos y Schnitzler, Ursula Storch

**Affiliations:** 1 Walther Straub Institute of Pharmacology and Toxicology, Ludwig Maximilian University of Munich, 80336 Munich, Germany; 2 Institute of Pharmacy, Clinical Pharmacy, University of Regensburg, 93053 Regensburg, Germany

**Keywords:** photopharmacology, photoswitchable activators, TRPC6 channels, current kinetics, PKC phosphorylation

## Abstract

Transient receptor potential canonical 6 (TRPC6) channels are promising drug targets for kidney, lung, and neurological diseases, making a detailed understanding of their regulation crucial to developing novel channel modulators with more precise modes of action. TRPC6 channels are commonly accepted as calcium-permeable, receptor-operated cation channels activated by diacylglycerol (DAG) downstream of phospholipase C (PLC) signaling. DAG, the endogenous activator of TRPC channels, also activates protein kinase C (PKC), which can phosphorylate TRPC6 and potentially modify its function. This study examined whether five putative PKC phosphorylation sites located in the C-terminus of TRPC6 affect channel gating. Using whole-cell patch-clamp recordings and utilizing photopharmacology with photoswitchable TRPC6 activators (OptoBI-1 and OptoDArG), we analyzed the activation, inactivation, and deactivation kinetics. Pharmacological modulation of PKC activity and strategic mutation of the phosphorylation sites—either to prevent or mimic phosphorylation—altered the current kinetics as well as the normalized slope conductances that were used to quantify differences in the curve progression of current–voltage relations, even when maximally induced current density amplitudes were unchanged. Our findings reveal activator-specific differences in TRPC6 current kinetics associated with C-terminal amino acid exchanges and PKC-dependent signaling, suggesting that phosphorylation-related mechanisms may fine-tune channel activity.

## 1. Introduction

TRPC6 channels belong to the family of transient receptor potential classical or canonical (TRPC) channels that are non-selective cation channels permeable for sodium, potassium and calcium ions. TRPC6 channels have various physiological and pathophysiological roles for the regulation of calcium influx in various cell types, such as podocytes, smooth muscle cells, neurons, and immune cells. Their dysfunction has been implicated in a growing number of diseases, highlighting their relevance as therapeutic targets (summarized in [1]). For example, patient mutations in the gene coding for TRPC6 are linked to familial forms of focal segmental glomerulosclerosis (FSGS), a severe kidney disorder characterized by proteinuria and progressive renal failure [2,3,4,5]. In the cardiovascular system, TRPC6 contributes to cardiac hypertrophy [6,7], vasoconstriction [8,9], and pulmonary arterial hypertension [10,11], and in the central nervous system, TRPC6 has been associated with neurodegenerative disorders, including Alzheimer’s disease [12]. Moreover, aberrant TRPC6 expression has been observed in cancer, and TRPC6 might be involved in tumor progression and metastasis [13]. Thus, TRPC6 channels are considered promising drug targets for the development of novel therapeutic interventions across multiple organ systems.

It is widely accepted that TRPC channels are receptor-operated channels which are activated downstream of G_q/11_-protein coupled receptors. This process involves activation of the phospholipase C which subsequently cleaves phosphoinositol-4,5-bisphosphate (PIP_2_) into the two second messengers inositol-1,4,5-trisphosphate (IP_3_) and diacylglycerol (DAG). DAG is an endogenous TRPC channel activator [14,15,16,17] that potentially binds in the pore region of the channel [18,19,20,21] resulting in channel opening and cation influx. In contrast to TRPC3, TRPC6, and TRPC7, TRPC4 and TRPC5 are not directly sensitive to DAG under basal conditions, because their DAG sensitivity is regulated by a C-terminal PKC phosphorylation site (T972 in TRPC5) located within the PDZ-binding motif “VTTRL”. This motif is conserved throughout the TRPC4/5 channel subgroup and facilitates interaction with the scaffolding protein Na^+^/H^+^ exchanger regulatory factor (NHERF) 1 and NHERF2 [22,23,24]. Dephosphorylation of T972 or amino acid exchange to alanine causes the dissociation of NHERF from the C-terminus, thereby rendering the channel directly sensitive to DAG [15,16,25]. Interestingly, T972 is also involved in channel inactivation during receptor-activation [26]. Amino acid exchange from threonine to alanine results in strongly decelerated current inactivation in the presence of carbachol [26]. This provides the first evidence that C-terminal PKC phosphorylation can alter current kinetics and might fine-tune channel activity.

PKC isoforms are known to phosphorylate serine or threonine residues, and some PKC isoforms can be directly activated by DAG (summarized in [27]). For instance, conventional (cPKCs) and novel PKCs (nPKCs) are both initially located in the cytosol in their inactive state. After DAG binding to the C1 domain of cPKCs and nPKCs, the enzymes are recruited from the cytosol to the plasma membrane, which results in the activation of the enzyme. In case of the cPKC isoforms α, βI, βII, and γ, activation also requires calcium binding to the C2 domain. Conversely, in nPKC isoforms δ, ε, η, and θ, DAG alone is sufficient for activation. It has been established that phosphorylation by PKC isoforms exerts a multitude of biological effects. Various studies have demonstrated the pivotal role of PKC-α in cell proliferation and differentiation [28]. PKC-ε has been shown to contribute to neuroprotection and cardiac function [29]. PKC-δ is involved in apoptosis [30] and immune responses [31]. PKC-θ has been identified as being essential for T-cell activation [32]. PKC-γ, which is predominantly expressed in neurons, and, in particular, in the cerebral cortex, hippocampus, and cerebellum, has been linked to synaptic plasticity and neurodegeneration [33]. PKC phosphorylation can take place in the consensus pattern “X-S/T-X-R/K” where X denotes any amino acid, S or T represents serine or threonine, and R or K the positively charged amino acids, arginine or lysine. However, until now, the interplay between PKC activity and TRPC channel regulation has still been largely elusive (summarized in [5]).

Nevertheless, some evidence suggests that PKC phosphorylation might influence TRPC channel activity. For instance, PKC phosphorylation of TRPC6 induced by the PKC activator phorbol 12-myristate 13 acetate (PMA) abolished receptor-operated TRPC6 channel activation [34] but had no effect on basal currents or currents induced by the membrane-permeable DAG-analog 1-oleoyl acetyl-*sn*-glycerol (OAG) [34], suggesting that receptor-operated TRPC6 channel activation is more complex and distinct from channel activation with DAG. PKC phosphorylation at position serine 768 in rat TRPC6, which corresponds to S768 in mouse and to S769 in human TRPC6, caused decreased TRPC6 activity and was required for the formation of a multiprotein complex containing TRPC6, muscarinic acetylcholine M1 receptor, and PKC [35]. Furthermore, phosphorylation of a non-canonical PKC phosphorylation site in TRPC6, serine at position 448 in mouse TRPC6, decreased the TRPC6 channel activity and reduced vasopressin-induced calcium influx in HEK293T and vascular smooth muscle A7r5 cells [36]. However, structural analysis by cryo-electron microscopy revealed that S448 is located in the transmembrane helix S1 [36], rendering this position inaccessible to the PKC. Furthermore, serine at position 814 in the C-terminal region of TRPC6 was found to be constitutively phosphorylated [37]. However, elimination of this phosphorylation site by amino acid exchange to alanine had no effect on TRPC6 channel activity. The enzyme responsible for phosphorylation of S814 is still elusive. Nevertheless, S814 in mouse TRPC6 (S815 in human TRPC6) occurred as constitutively phosphorylated in high throughput screenings, for example, in spleen [38,39,40], lung [38,40], thymus [40], blood [40], and colorectal cancer samples [41] and in non-small cell lung cancer tumors [42] in patients, suggesting that S814 might be important for the regulation of TRPC6 channel activity.

In addition, there is evidence that C-terminal PKC phosphorylation of the closely related TRPC3 and TRPC7 channels also alters their function. For example, PKC phosphorylation of serine at position 712 of human TRPC3, which corresponds to S768 in mouse TRPC6 and S714 in mouse TRPC7, is located in the C-terminus and was shown to decrease OAG-mediated channel activity [43]. A further potential PKC phosphorylation site of TRPC3 was identified at position threonine 573. This site is located in the cytoplasmic S4-S5 linker region which enhances the formation of a TRPC3-calcineurin complex leading to activation of the transcription factor NFAT [44]. However, no effect of PKC phosphorylation of T573 on TRPC3 channel activity was reported. In addition, PKC phosphorylation of serine 714 in mouse TRPC7 was associated with decreased TRPC7 channel activity, which was linked to changes in the cytoskeletal organization of myofibroblasts [45]. In summary, there is preliminary evidence that PKC phosphorylation can alter TRPC3/6/7 channel activity, but the precise effects of PKC phosphorylation on the current kinetics is still elusive. Furthermore, PKC phosphorylation increased TRPC1 channel activity [46,47,48,49,50], resulting in enhanced endothelial cell permeability [46,48], PIP_2_-mediated store-operated calcium influx [47], and increased insulin secretion [50]. Nevertheless, specific PKC phosphorylation sites in TRPC1 require further investigation. Altogether, although there is initial evidence that PKC phosphorylation can influence TRPC channel activity, the role of C-terminal PKC phosphorylation and dephosphorylation in TRPC current kinetics remains to be elucidated.

We previously demonstrated that photopharmaceuticals targeting TRP channels allow for a reliable determination of the activation, deactivation and inactivation kinetics of whole-cell currents [51,52], since, using photoswitchable activators, the wash-in and wash-out effects that usually occur when compounds are applied via bath solutions are circumvented. The basis for photoswitching is the insertion of a photoswitchable moiety [21,52,53,54,55] that enables light-induced *cis*- or *trans*-isomerization by illumination with UV or blue light, respectively. Photoswitchable drugs are high-precision tools that allow for spatiotemporal control of channel function [21,52,53,54,56,57]. At present, two photoswitchable DAG derivatives—PhoDAG and OptoDArG—and the photoswitchable TRPC3/6 channel activator OptoBI-1, that is based on the non-lipidic activator GSK 1702934A, are available to control the TRPC6 channel function [21,53,54,56,57].

Despite the established roles of PKC and TRPC6 in diverse pathologies, how PKC-dependent phosphorylation shapes TRPC6 gating kinetics—particularly under defined and physiologically relevant activation modes—remains unresolved. Using TRPC6 variants in which PKC phosphorylation sites were replaced either by glutamate or aspartate to approximate phosphorylation-induced negative charge or by alanine to prevent phosphorylation, in combination with rapid and reversible photopharmacological activation, we provide new insight into how specific PKC sites fine-tune channel behavior. This approach not only dissects a fundamental aspect of TRPC6 regulation, but also highlights mechanistic differences that may be exploited in the rational design of TRPC6-targeted therapies.

## 2. Results

### 2.1. Amino Acid Exchanges of Serine 768 Result in Distinct Current Kinetics

Using PrositeScan (motif PS00005) with the PKC consensus motif “X-S/T-X-R/K”, 14 putative PKC phosphorylation sites in mouse TRPC6 were identified. Seven sites were located at the N-terminus (S4, T9, S14, T23, S55, T64 and S258), from which six were positioned proximally (S4, T9, S14, T23, S55 and T64) and one distally (S258) to the ankyrin repeat domain, and one was located extracellularly (T562) which rendered it inaccessible to intracellular PKC isoforms. T629 was located at the beginning of the TM5, thereby creating a possibility for PKC phosphorylation, while T673 was located in the pore helix, making PKC phosphorylation unlikely. However, four PKC phosphorylation sites were located at the C-terminus (S768, S835, S892, and S928). Serine 768 was located directly after the TRP domain, preceding the horizontal helix with the calmodulin and IP_3_ receptor-binding (CIRB) domain. Serine 835 was positioned between the TRP domain and the horizontal helix with the CIRB domain but closer to the calmodulin (CaM)-binding domain. Serine 892 was located in the structurally more elucidated vertical helix with the coiled-coil domain perpendicular to the cytoplasmic membrane surface at the distal end of the C-terminus which makes it unlikely that this amino acid is phosphorylated by PKC isoforms. Serine 928 was located at the structurally non-elucidated and far distal end of the C-terminus following the vertical helix with the coiled-coil domain.

The four C-terminally located putative PKC phosphorylation sites were selected for amino acid exchanges either to alanine, to prevent phosphorylation, or to aspartate and glutamate, which are negatively charged amino acids that were utilized to mimic permanent phosphorylation [58,59]. It should be noted that substitutions with aspartate or glutamate represent pseudo-phosphorylation rather than true phosphorylation, and therefore only approximate the electrostatic consequences of phosphorylation without reproducing its full structural context. Furthermore, despite not representing a PKC phosphorylation site, serine 814, which is located before the horizontal helix with the CIRB domain, was selected for further analysis, since S814 in TRPC6 was constitutively phosphorylated in several pathological states [37,38,39,40,41,42] and thus might alter the TRPC6 channel activity. The positions of the four C-terminal PKC phosphorylation sites and of S814 are displayed in Supplemental Figure S1.

**Figure 1:**
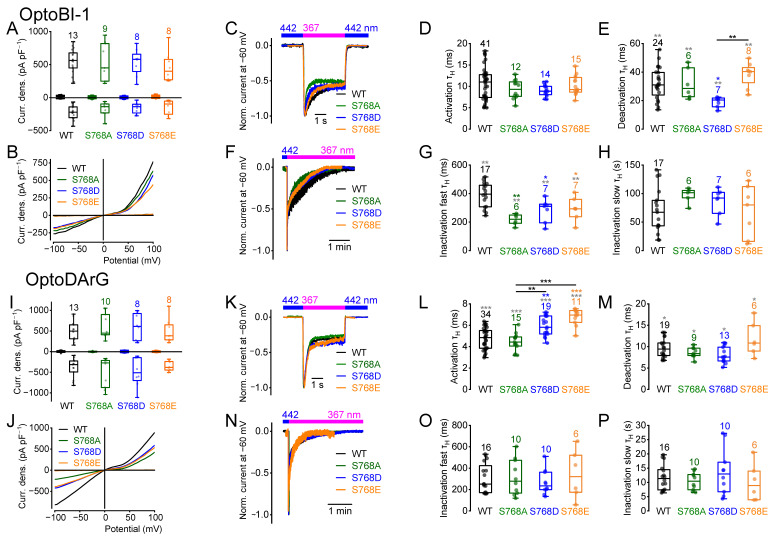
Amino acid exchanges of serine 768 result in distinct current kinetics. Electrophysiological whole-cell measurements of HEK293T cells overexpressing TRPC6 and indicated TRPC6 mutants in the presence of 10 µM OptoBI-1 (**A**–**H**) or 30 µM OptoDArG (**I**–**P**). (**A**,**I**) Summaries of current densities (‘Curr. dens.’) at potentials of ±100 mV evoked by light. First small boxplots represent current densities in the presence of blue light (442 nm) which establishes *trans*-configuration and second boxplots represent maximal current densities in the presence of UV light (367 nm) which establishes *cis*-configuration. (**A**) No significant differences were observed between *trans-*OptoBI-1-induced or *cis*-OptoBI-1-induced current densities of the mutant channels compared to the wildtype. (**I**) *trans*-OptoDArG- or *cis*-OptoDArG-induced current densities of the mutant channels were not significantly different compared to the wildtype. (**B**,**J**) Representative current density–voltage relations induced by illumination with UV light. (**C**,**F**,**K**,**N**) Representative normalized current time courses of inward currents at constant holding potential of -60 mV during photoswitching from blue light (blue bar) to UV light (magenta bar) (**F**,**N**) and back to blue light (blue bar) (**C**,**K**). (**D**,**E**,**L**,**M**) Summaries of half-life time constants (τ_H_) of the activation (**D**,**L**) and deactivation (**E**,**M**) kinetics. (**G**,**H**,**O**,**P**) Summaries of half-life time constants (τ_H_) of the fast (**G**,**O**) and slow (**H**,**P**) inactivation kinetics. (**D**,**E**,**G**,**H**,**L**,**M**,**O**,**P**) Data are displayed as boxplots and interquartile ranges. Numbers over boxplots indicate number of measured cells. Statistical significance was determined using the Kruskal–Wallis test with Dunn’s post hoc analysis. Gray asterisks indicate overall differences among all groups identified by the Kruskal–Wallis test, whereas colored and black asterisks indicate pairwise differences revealed by Dunn’s post hoc analysis (colored vs. wild type; black between the groups connected by horizontal lines). * *p* < 0.05; ** *p* < 0.01; *** *p* < 0.001.

To elucidate whether amino acid exchange of S786 influences activation, deactivation, or inactivation kinetics, we performed electrophysiological whole-cell measurements (Figure 1). To determine the current kinetics, we employed photopharmacology with OptoBI-1 and OptoDArG, wherein photoswitching was induced by illumination with high intensity and fast switching LEDs [51,57]. The *cis*-configuration was established by applying UV light, and the *trans*-configuration by applying blue light. Switching from blue light to UV light resulted in rapid TRPC6 current increases. OptoBI-1 and OptoDArG were applied in their maximally effective concentrations. In the presence of OptoBI-1 (10 µM), the maximally induced current densities during UV light illumination were not significantly different between the three TRPC6 mutants and the wild type (Figure 1A,B). The current kinetics were obtained from inward currents measured at a constant holding potential of −60 mV [51], which represents a compromise between physiological relevance and recording stability. At this potential, cells could be maintained for extended periods without leak increase or rundown, while inward currents remained large enough for reliable kinetic analysis. Moreover, −60 mV approximates the resting potential of many native cell types expressing TRPC6 channels, ensuring that the measured kinetics reflect physiologically relevant channel behavior. Switching from blue to UV light resulted in fast current activation (Figure 1C,D), followed by a biphasic inactivation, which comprised a fast and a slow component of inactivation (Figure 1F–H and Supplemental Figures S2 and S3). Switching from UV to blue light caused rapid deactivation (Figure 1C,E). Current kinetics were determined by calculating the respective half-life time constants (τ_H_) for activation, deactivation, and fast and slow inactivation [51,52,57,60] (Supplemental Figure S3). The activation kinetics of all three mutants and wildtype TRPC6 were not significantly different (Figure 1D). In contrast, measuring untransfected HEK293T cells, no current responses were observed during photoswitching in the presence of OptoBI-1 or OptoDArG (Supplemental Figure S4). However, the mutant S768D showed significantly faster deactivation kinetics than the wildtype and the S768E mutant (Figure 1E). The fast inactivation kinetics of all three mutants in the presence of UV light were significantly accelerated (Figure 1G), but the slow inactivation kinetics were unchanged (Figure 1H). Using the DAG derivative OptoDArG (30 µM) for photoswitching, the maximally induced current densities of wildtype TRPC6 and the mutants were also not significantly different (Figure 1I,J). However, when analyzing the activation kinetics, the mutants S768D and S768E showed significantly slower activation kinetics compared to the wildtype and to the mutant S768A (Figure 1L). In contrast to what was observed in the presence of OptoBI-1, the deactivation kinetics of the mutant S768D did not differ significantly from the wildtype (Figure 1M), although the overall comparison among all groups revealed a significant difference in the Kruskal–Wallis test. Altogether, these findings suggest activator-specific effects on the current kinetics. Interestingly, kinetic analysis revealed that the mutant S768D, but not S768E, displayed faster deactivation kinetics (Figure 1E). However, in the presence of OptoDArG, both mutants showed slower activation kinetics (Figure 1L). Interestingly, only in the presence of OptoBI-1, but not in the presence of OptoDArG (Figure 1O), amino acid exchanges resulted in enhanced fast inactivation kinetics (Figure 1G). The slow inactivation kinetics in the presence of OptoBI-1 and OptoDArG were unchanged (Figure 1H,P).

Furthermore, we calculated the normalized slope conductance (NSC) from the current density–voltage curves selected at maximally induced current densities, since calculation of the NSC allows for a quantitative comparison of the current density–voltage curves over a wide potential range from −100 up to +100 mV [61]. Analyzing the NSC curve progressions of the mutants S768A, S768D and S768E compared to the wildtype, we found that in the presence of OptoBI-1 all three mutants showed significant differences in their NSC curve progression at positive potentials (Supplemental Figure S5A). However, using OptoDArG, the NSC curves were similar to the wildtype (Supplemental Figure S5B), suggesting that both activators cause distinct channel-gating behavior.

### 2.2. Amino Acid Exchanges of Serine 814 Influence the Current Kinetics

**Figure 2:**
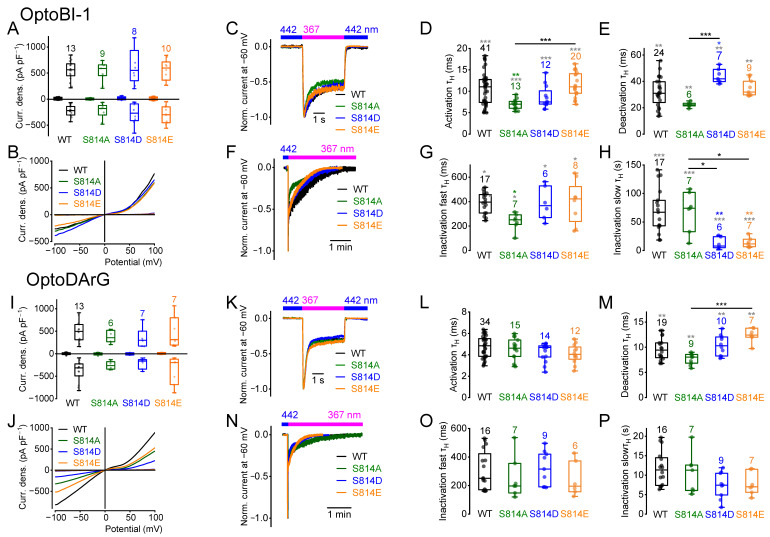
Amino acid exchanges of serine 814 influence the current kinetics. Electrophysiological whole-cell measurements of HEK293T cells overexpressing TRPC6 and indicated TRPC6 mutants in the presence of 10 µM OptoBI-1 (**A**–**H**) or 30 µM OptoDArG (**I**–**P**). (**A**,**I**) Summaries of current densities (‘Curr. dens.’) at potentials of ±100 mV evoked by light. First small boxplots represent current densities in the presence of blue light which establishes *trans*-configuration, and second boxplots represent maximal current densities in the presence of UV light which establishes *cis*-configuration. (**A**) No significant differences were observed between *trans-*OptoBI-1-induced or *cis*-OptoBI-1-induced current densities of the mutant channels compared to the wildtype. (**I**) *trans*-OptoDArG- or *cis*-OptoDArG-induced current densities of the mutant channels were not significantly different compared to the wildtype. (**B**,**J**) Representative current-density–voltage relations induced by illumination with UV light. (**C**,**F**,**K**,**N**) Representative normalized current time courses of inward currents at constant holding potential of −60 mV during photoswitching from blue light (blue bar) to UV light (magenta bar) (**F**,**N**) and back to blue light (blue bar) (**C**,**K**). (**D**,**E**,**L**,**M**) Summaries of half-life time constants (τ_H_) of the activation (**D**,**L**) and deactivation (**E**,**M**) kinetics. (**G**,**H**,**O**,**P**) Summaries of half-life time constants (τ_H_) of the fast (**G**,**O**) and slow (**H**,**P**) inactivation kinetics. (**D**,**E**,**G**,**H**,**L**,**M**,**O**,**P**) Data are displayed as boxplots and interquartile ranges. Numbers over boxplots indicate number of measured cells. Gray asterisks indicate overall differences among all groups identified by the Kruskal–Wallis test, whereas colored and black asterisks indicate pairwise differences revealed by Dunn’s post hoc analysis (colored vs. wild type; black between the groups connected by horizontal lines). * *p* < 0.05; ** *p* < 0.01; *** *p* < 0.001.

Next, we analyzed S814, which is constitutively phosphorylated in various disease states. Illumination with UV light in the presence of OptoBI-1 and OptoDArG resulted in maximally induced current densities that were not significantly different (Figure 2A,B,I,J), which is in line with previous findings [37]. Analyzing the current kinetics, the *cis*-OptoBI-1-induced currents of the mutant S814A showed a significantly accelerated activation kinetics and fast inactivation kinetics (Figure 2D,G). However, the deactivation kinetics of S814D, but not of S814E, were significantly slower compared to the wildtype and S814A (Figure 2E). The slow inactivation kinetics of S814A were unchanged (Figure 2H). The slow inactivation kinetics of S814D and S814E were significantly faster compared to the wildtype and to S814A (Figure 2H). Analyzing the mutants S814D and S814E in the presence of OptoDArG (Figure 2P), the slow inactivation kinetics were unchanged. The OptoDArG-induced current kinetics of S814A were not significantly different (Figure 2L,M,O,P). In the presence of OptoDArG, the mutant S814E, but not S814D, exhibited significantly slower deactivation kinetics compared to S814A (Figure 2M) while in the presence of OptoBI-1, the deactivation kinetics of S814D, and not of S814E, were significantly faster compared to S814A (Figure 2E) indicating that the two pseudo-phosphomimetic amino acids have different effects on the deactivation kinetics. These findings again suggest compound-specific effects on the current kinetics of the amino acid exchanges at position S814.

The NSC calculation of the *cis*-OptoBI-1-induced current-density–voltage relations of the mutant S814A exhibited significant differences in the NSC curve progression only at positive potentials (Supplemental Figure S6). The NSC curve progressions of the *cis*-OptoDArG-induced currents were unchanged. Altogether, phosphorylation at S814 influenced the current kinetics without changing the maximally induced current density amplitudes. The slower deactivation and/or a faster slow inactivation and changes in the NSC curve progression suggested an effect on the channel-gating behavior, which might contribute to the pathophysiological states in which this phosphorylation site occurs.

### 2.3. Amino Acid Exchanges of Serine 835 Alter the Current Kinetics

**Figure 3:**
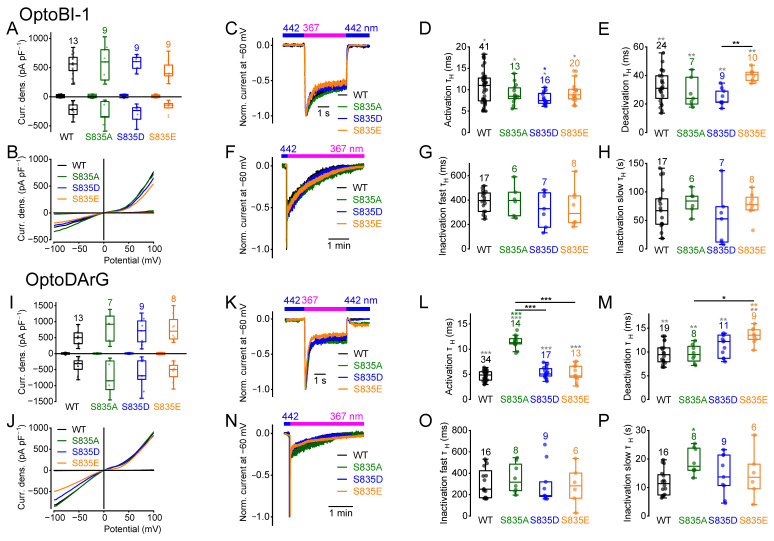
Amino acid exchanges of serine 835 alter the current kinetics. Electrophysiological whole-cell measurements of HEK293T cells overexpressing TRPC6 and indicated TRPC6 mutants in the presence of 10 µM OptoBI-1 (**A**–**H**) or 30 µM OptoDArG (**I**–**P**). (**A**,**I**) Summaries of current densities (‘Curr. dens.’) at potentials of ±100 mV evoked by light. First small boxplots represent current densities in the presence of blue light) which establishes *trans*-configuration and second boxplots represent maximal current densities in the presence of UV light which establishes *cis*-configuration. (**A**) No significant differences were observed between *trans-*OptoBI-1-induced or *cis*-OptoBI-1-induced current densities of the mutant channels compared to the wildtype. (**I**) *trans*-OptoDArG- or *cis*-OptoDArG-induced current densities of the mutant channels were not significantly different compared to the wildtype. (**B**,**J**) Representative current-density–voltage relations induced by illumination with UV light. (**C**,**F**,**K**,**N**) Representative normalized current time courses of inward currents at constant holding potential of -60 mV during photoswitching from blue light (blue bar) to UV light (magenta bar) (**F**,**N**) and back to blue light (blue bar) (**C**,**K**). (**D**,**E**,**L**,**M**) Summaries of half-life time constants (τ_H_) of the activation (**D**,**L**) and deactivation (**E**,**M**) kinetics. (**G**,**H**,**O**,**P**) Summaries of half-life time constants (τ_H_) of the fast (**G**,**O**) and slow (**H**,**P**) inactivation kinetics. (**D**,**E**,**G**,**H**,**L**,**M**,**O**,**P**) Data are displayed as boxplots and interquartile ranges. Numbers over boxplots indicate number of measured cells. Gray asterisks indicate overall differences among all groups identified by the Kruskal–Wallis test, whereas colored and black asterisks indicate pairwise differences revealed by Dunn’s post hoc analysis (colored vs. wild type; black between the groups connected by horizontal lines). * *p* < 0.05; ** *p* < 0.01; *** *p* < 0.001.

The amino acid exchange of serine at position 835 to alanine, aspartate, or glutamate resulted in functional channels with comparable maximally induced current densities (Figure 3A,B,I,J). Analysis of the current kinetics revealed significantly slower activation kinetics of the mutant S835A in the presence of OptoDArG but not of OptoBI-1 (Figure 3D,L). However, in the presence of OptoBI-1, the activation kinetics of the mutant S835D were significantly faster (Figure 3D). Moreover, OptoDArG induced significantly slower deactivation kinetics of the mutant S835E compared to the wildtype and to S835A (Figure 3M). OptoBI-1-induced deactivation kinetics showed significant differences between all groups and between S835D and S835E (Figure 3E). The fast inactivation kinetics in the presence of OptoBI-1 or OptoDArG were unchanged (Figure 3G,O). The slow inactivation kinetics of the OptoDArG-induced currents of the mutant S835A were significantly slower compared to the wildtype (Figure 3P). Altogether, although the maximally induced current densities were unchanged, OptoBI-1 and OptoDArG elicited distinct compound-specific changes in the current kinetics. In addition, regarding the activation and deactivation kinetics, the pseudo-phosphomimetic mutants did not behave in the same way in the presence of OptoBI-1 (Figure 3D,E).

Calculation of the NSC curves of the maximal *cis*-OptoBI-1-induced current-density–voltage relations revealed only slight differences in the NSC curve progression of the mutant S835E compared to the wildtype at positive potentials (Supplemental Figure S7A). The NSC curve progressions of the *cis*-OptoDArG-induced currents were unchanged (Supplemental Figure S7A).

### 2.4. Amino Acid Exchanges of Serine 892 Influence Current Densities and Current Kinetics

**Figure 4:**
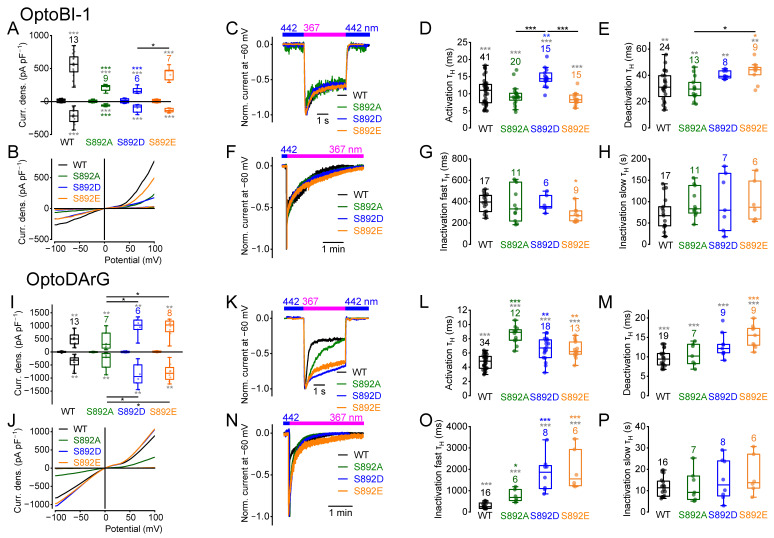
Amino acid exchanges of serine 892 influence current densities and current kinetics. Electrophysiological whole-cell measurements of HEK293T cells overexpressing TRPC6 and indicated TRPC6 mutants in the presence of 10 µM OptoBI-1 (**A**–**H**) or 30 µM OptoDArG (**I**–**P**). (**A**,**I**) Summaries of current densities (‘Curr. dens.’) at potentials of ±100 mV evoked by light. First small boxplots represent current densities in the presence of blue light which establishes *trans*-configuration, and second boxplots represent maximal current densities in the presence of UV light which establishes *cis*-configuration. (**A**) Significant differences were observed between *cis-*OptoBI-1-induced current densities of the mutants S892A and S892D compared to the wildtype. (**I**) *cis*-OptoDArG-induced current densities of the mutants S892D and S892E were significantly different compared to the wildtype. (**B**,**J**) Representative current-density–voltage relations induced by illumination with UV light. (**C**,**F**,**K**,**N**) Representative normalized current time courses of inward currents at constant holding potential of −60 mV during photoswitching from blue light (blue bar) to UV light (magenta bar) (**F**,**N**) and back to blue light (blue bar) (**C**,**K**). (**D**,**E**,**L**,**M**) Summaries of half-life time constants (τ_H_) of the activation (**D**,**L**) and deactivation (**E**,**M**) kinetics. (**G**,**H**,**O**,**P**) Summaries of half-life time constants (τ_H_) of the fast (**G**,**O**) and slow (**H**,**P**) inactivation kinetics. (**D**,**E**,**G**,**H**,**L**,**M**,**O**,**P**) Data are displayed as boxplots and interquartile ranges. Numbers over boxplots indicate number of measured cells. Gray asterisks indicate overall differences among all groups identified by the Kruskal–Wallis test, whereas colored and black asterisks indicate pairwise differences revealed by Dunn’s post hoc analysis (colored vs. wild type; black between the groups connected by horizontal lines). * *p* < 0.05; ** *p* < 0.01; *** *p* < 0.001.

Next, we analyzed the role of serine 892 on current density amplitudes and current kinetics. As mentioned, phosphorylation of S892 seems unlikely because of its poor accessibility for the PKC. However, amino acid exchanges from serine 892 to alanine and aspartate resulted in significantly reduced *cis*-OptoBI-1-induced current densities at +100 mV compared to the wildtype, while the exchange to glutamate had no effect (Figure 4A,B). However, the maximally induced current densities at +100 mV of S892D and S892E were significantly different (Figure 4A). In contrast, the *cis*-OptoDArG-induced current densities of the mutants S892A and S892D were not significantly different to the wildtype (Figure 4I,J), but compared to S892A, the maximally induced current densities of S892D and S892E were significantly increased (Figure 4I). The half-life time constants for the activation kinetics of the mutant S892D induced by *cis*-OptoBI-1 were higher (Figure 4D) compared to the wildtype and to the mutants S892A and S892E. The mutant S892E caused significantly slower deactivation kinetics induced by OptoBI-1 or OptoDArG compared to the wildtype (Figure 4E,M). In contrast to OptoBI-1, all three amino acid exchanges resulted in slower OptoDArG-induced activation kinetics (Figure 4L). In addition, the OptoBI-1-induced fast inactivation kinetics of the mutant S892E were significantly faster compared to the wildtype (Figure 4G). However, OptoDArG-induced fast inactivation kinetics of the mutants were significantly slower compared to the wildtype (Figure 4O). Altogether, each TRPC6 activator elicited specific effects on the current kinetics of distinct mutants.

Calculation of the NSC curve progressions induced by *cis*-OptoBI-1 revealed significant differences, mainly at positive potentials in the case of the mutant S892E (Supplemental Figure S8A). However, the NSC curve progressions of *cis*-OptoDArG-induced current–voltage relations showed significant differences in all three mutants (Supplemental Figure S8B). To summarize, preventing or mimicking phosphorylation by amino acid exchanges influenced current density amplitudes as well as current kinetics in an activator-specific manner, suggesting that serine 892 and its phosphorylation state might fine-tune the channel activity.

### 2.5. Amino Acid Exchanges of Serine 928 Cause Changes in the Current Kinetics

**Figure 5:**
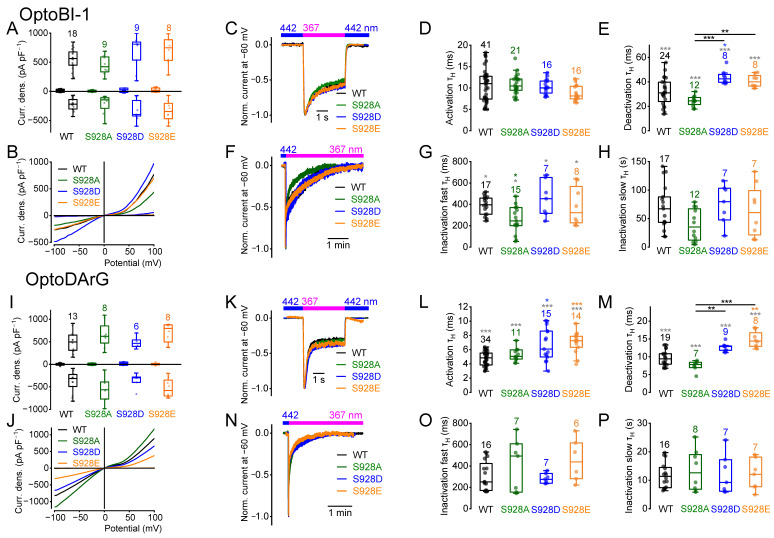
Amino acid exchanges of serine 928 cause changes in the current kinetics. Electrophysiological whole-cell measurements of HEK293T cells overexpressing TRPC6 and indicated TRPC6 mutants in the presence of 10 µM OptoBI-1 (**A**–**H**) or 30 µM OptoDArG (**I**–**P**). (**A**.**I**) Summaries of current densities (‘Curr. dens.’) at potentials of ±100 mV evoked by light. First small boxplots represent current densities in the presence of blue light, which establishes *trans*-configuration, and second boxplots represent maximal current densities in the presence of UV light, which establishes *cis*-configuration. (**A**) No significant differences were observed between *trans-*OptoBI-1-induced or *cis*-OptoBI-1-induced current densities of the mutant channels compared to the wildtype. (**I**) *trans*-OptoDArG- or *cis*-OptoDArG-induced current densities of the mutant channels were not significantly different compared to the wildtype. (**B**,**J**) Representative current-density–voltage relations induced by illumination with UV light. (**C**,**F**,**K**,**N**) Representative normalized current time courses of inward currents at constant holding potential of -60 mV during photoswitching from blue light (blue bar) to UV light (magenta bar) (**F**,**N**) and back to blue light (blue bar) (**C**,**K**). (**D**,**E**,**L**,**M**) Summaries of half-life time constants (τ_H_) of the activation (**D**,**L**) and deactivation (**E**,**M**) kinetics. (**G**,**H**,**O**,**P**) Summaries of half-life time constants (τ_H_) of the fast (**G**,**O**) and slow (**H**,**P**) inactivation kinetics. (**D**,**E**,**G**,**H**,**L**,**M**,**O**,**P**) Data are displayed as boxplots and interquartile ranges. Numbers over boxplots indicate number of measured cells. Gray asterisks indicate overall differences among all groups identified by the Kruskal–Wallis test, whereas colored and black asterisks indicate pairwise differences revealed by Dunn’s post hoc analysis (colored vs. wild type; black between the groups connected by horizontal lines). * *p* < 0.05; ** *p* < 0.01; *** *p* < 0.001.

S928 was positioned two amino acids proximal to the C-terminal end of the TRPC6 protein. Thus, we aimed to analyze whether amino acid exchange at this position might have similar effects on the inactivation kinetics as T972 in TRPC5, which lies three amino acids proximal to the C-terminal end [26]. The maximal *cis*-OptoBI-1- and *cis*-OptoDArG-induced current densities were comparable to the wildtype and to each other (Kruskal–Wallis test) (Figure 5A,B,I,J). However, while the OptoBI-1-induced activation kinetics were unchanged (Figure 5D), the OptoDArG-induced activation kinetics of the mutants S928E and S928D were significantly slower compared to the wildtype (Figure 5L). OptoBI-1 induced slower deactivation kinetics of the mutant S928D (Figure 5E), and OptoDArG of the mutant S928E compared to the wildtype (Figure 5M). The deactivation kinetics of S928D and S928E were significantly slower compared to S928A (Figure 5E,M). The OptoBI-1-induced fast inactivation kinetics of the mutant S928A were significantly faster (Figure 5G), and the slow inactivation kinetics were unchanged (Figure 5H). In addition, the OptoDArG-induced fast and slow inactivation kinetics were unchanged (Figure 5O,P).

OptoBI-1-induced changes in the fast inactivation kinetics of the mutant S928A were reflected by changes in the NSC curve progression at positive potentials (Supplemental Figure S9A). In case of OptoDArG-induced currents, only slight changes in the NSC curve progressions at negative potentials were observed for the mutants S928A and S928E (Supplemental Figure S9B). Altogether, although the maximally induced current density amplitudes were unchanged, amino acid exchanges at serine 928 influenced the current kinetics in a compound-specific manner suggesting different channel-gating behavior. Interestingly, the mutant S928A showed enhanced fast inactivation kinetics in the presence of OptoBI-1, suggesting that this amino acid exchange accelerates, rather than decelerating, the inactivation kinetics. However, OptoDArG-induced inactivation kinetics were completely unaffected. To summarize, our findings suggest a different role of S928 for TRPC6 channel gating than T972 for TRPC5.

### 2.6. Amino Acid Exchange from Serine 928 to Glycine and C-Terminal Truncations Influence the Current Kinetics

**Figure 6:**
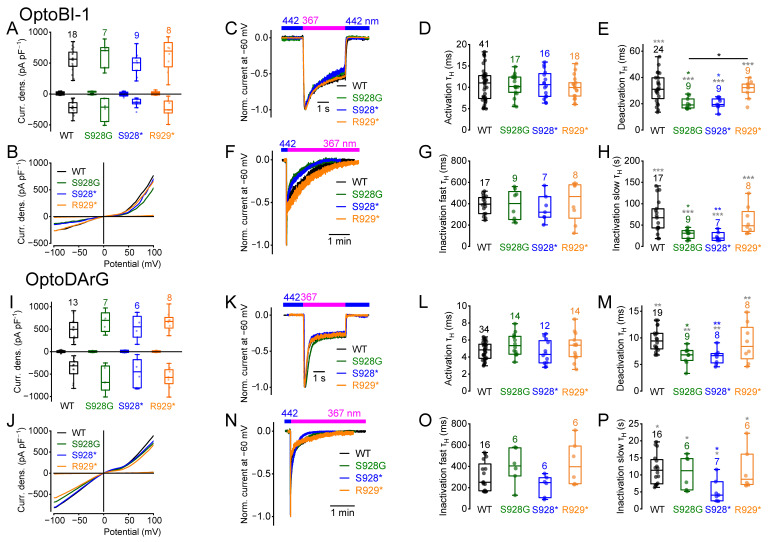
Amino acid exchange from serine 928 to glycine and C-terminal truncations influence the current kinetics. Electrophysiological whole-cell measurements of HEK293T cells overexpressing TRPC6 and indicated TRPC6 mutants in the presence of 10 µM OptoBI-1 (**A**–**H**) or 30 µM OptoDArG (**I**–**P**). (**A**,**I**) Summaries of current densities (‘Curr. dens.’) at potentials of ±100 mV evoked by light. First small boxplots represent current densities in the presence of blue light, which establishes *trans*-configuration, and second boxplots represent maximal current densities in the presence of UV light, which establishes *cis*-configuration. (**A**) No significant differences were observed between *trans-*OptoBI-1-induced or *cis*-OptoBI-1-induced current densities of the mutant channels compared to the wildtype. (**I**) *trans*-OptoDArG- or *cis*-OptoDArG-induced current densities of the mutant channels were not significantly different compared to the wildtype. (**B**,**J**) Representative current-density–voltage relations induced by illumination with UV light. (**C**,**F**,**K**,**N**) Representative normalized current time courses of inward currents at constant holding potential of -60 mV during photoswitching from blue light (blue bar) to UV light (magenta bar) (**F**,**N**) and back to blue light (blue bar) (**C**,**K**). (**D**,**E**,**L**,**M**) Summaries of half-life time constants (τ_H_) of the activation (**D**,**L**) and deactivation (**E**,**M**) kinetics. (**G**,**H**,**O**,**P**) Summaries of half-life time constants (τ_H_) of the fast (**G**,**O**) and slow (**H**,**P**) inactivation kinetics. (**D**,**E**,**G**,**H**,**L**,**M**,**O**,**P**) Data are displayed as boxplots and interquartile ranges. Numbers over boxplots indicate number of measured cells. Gray asterisks indicate overall differences among all groups identified by the Kruskal–Wallis test, whereas colored and black asterisks indicate pairwise differences revealed by Dunn’s post hoc analysis (colored vs. wild type; black between the groups connected by horizontal lines). * *p* < 0.05; ** *p* < 0.01; *** *p* < 0.001.

We next analyzed whether amino acids exchange to glycine and C-terminal truncations of TRPC6 through the insertion of stop codons at positions S928 (S928*) or R929 (R929*) to eliminate the last two or three amino acids, respectively, and also alter the current kinetics. Amino acid exchange to glycine and the insertion of stop codons had no effect on the maximally induced current densities in the presence of *cis*-OptoBI-1 or *cis*-OptoDArG compared to the wildtype (Figure 6A,B,I,J). However, the OptoBI-1 and OptoDArG-induced deactivation kinetics of the mutant S928G were altered (Figure 6E,M). The slow OptoBI-1-induced inactivation kinetics were also enhanced (Figure 6H). However, S928G had no effect on OptoBI-1-induced fast inactivation kinetics (Figure 6G). Furthermore, the deactivation kinetics of S928G were significantly faster compared to R929* (Figure 6E). OptoBI-1- and OptoDArG-induced deactivation kinetics of the truncated mutant S928* were also significantly faster (Figure 6D,G,L,O). Furthermore, OptoBI-1- and OptoDArG-induced slow inactivation kinetics of the mutant S928* were significantly enhanced (Figure 6H,P). However, the current kinetics of the mutant R929* were not significantly different compared to the wildtype channel, suggesting that the elimination of the last two amino acids has no effect on channel gating. In contrast, elimination of the last three amino acids accelerated deactivation and slow inactivation kinetics independently of the applied activator.

The NSC curve progressions calculated from the *cis*-OptoBI-1-induced current–voltage relations of the mutants S928G, S928*, and R929* were not different from the wildtype (Supplemental Figure S10A). In case of *cis*-OptoDArG-induced current–voltage relations, only slight differences in the NSC curve progression were found at negative potentials in the mutant S928* (Supplemental Figure S10B). Altogether, C-terminal truncation of TRPC6 had a more pronounced effect on channel kinetics than substitution of serine at the putative PKC phosphorylation site.

### 2.7. Multiple Amino Acid Exchanges Influence the Current Kinetics

**Figure 7:**
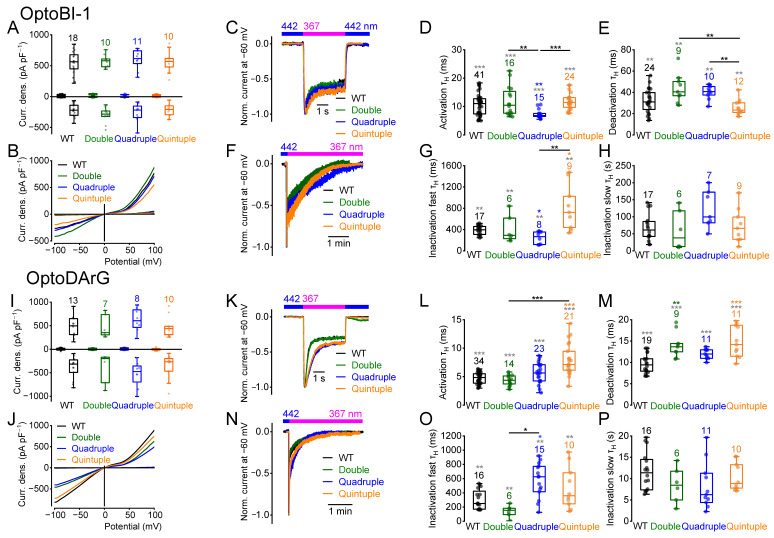
Multiple amino acid exchanges influence the current kinetics. Electrophysiological whole-cell measurements of HEK293T cells overexpressing TRPC6 or TRPC6 double (S814A and S835A), quadruple (S768A, S814A, S835A, and S892A), or quintuple (S768A, S814A, S835A, S892A, and S928A) mutants in the presence of 10 µM OptoBI-1 (**A**–**H**) or 30 µM OptoDArG (**I**–**P**). (**A**,**I**) Summaries of current densities (‘Curr. dens.’) at potentials of ±100 mV evoked by light. First small boxplots represent current densities in the presence of blue light, which establishes *trans*-configuration, and second boxplots represent maximal current densities in the presence of UV light, which establishes *cis*-configuration. (**A**) No significant differences were observed between *trans-*OptoBI-1-induced or *cis*-OptoBI-1-induced current densities of the mutant channels compared to the wildtype. (**I**) *trans*-OptoDArG- or *cis*-OptoDArG-induced current densities of the mutant channels were not significantly different compared to the wildtype. (**B**,**J**) Representative current-density–voltage relations induced by illumination with UV light. (**C**,**F**,**K**,**N**) Representative normalized current time courses of inward currents at constant holding potential of -60 mV during photoswitching from blue light (blue bar) to UV light (magenta bar) (**F**,**N**) and back to blue light (blue bar) (**C**,**K**). (**D**,**E**,**L**,**M**) Summaries of half-life time constants (τ_H_) of the activation (**D**,**L**) and deactivation (**E**,**M**) kinetics. (**G**,**H**,**O**,**P**) Summaries of half-life time constants (τ_H_) of the fast (**G**,**O**) and slow (**H**,**P**) inactivation kinetics. (**D**,**E**,**G**,**H**,**L**,**M**,**O**,**P**) Data are displayed as boxplots and interquartile ranges. Numbers over boxplots indicate number of measured cells. Gray asterisks indicate overall differences among all groups identified by the Kruskal–Wallis test, whereas colored and black asterisks indicate pairwise differences revealed by Dunn’s post hoc analysis (colored vs. wild type; black between the groups connected by horizontal lines). * *p* < 0.05; ** *p* < 0.01; *** *p* < 0.001.

We next performed double, quadruple, and quintuple amino acid exchanges to alanine at potential PKC phosphorylation sites. We started with amino acid exchanges from serine to alanine at the positions S814 and S835, as their single mutations had already led to significant changes in current kinetics (Figure 2 and Figure 3). The double mutant was still functional, showing maximally induced current densities similar to the wildtype (Figure 7A,B,I,J). However, OptoDArG-induced deactivation kinetics were significantly slower (Figure 7M). Thus, the double mutant exhibits slight changes in current kinetics and shows no additive effects of the single mutations.

Next, the quadruple mutant was analyzed, which encompassed amino acid exchanges from serine to alanine at positions 768, 814, 835, and 892. The mutant was still functional, showing similar maximally induced current densities as the wildtype (Figure 7A,B,I,J). The quadruple mutant exhibited faster OptoBI-1-induced activation and fast inactivation kinetics (Figure 7D,G), but OptoDArG-induced activation kinetics were comparable to the wildtype, and fast inactivation kinetics were significantly slower (Figure 7L,O). OptoBI-1- and OptoDArG-induced deactivation and slow inactivation kinetics were not significantly different from the wildtype (Figure 7E,H,M,P). Altogether, amino acid substitutions at all four putative PKC phosphorylation sites markedly affected current kinetics in an activator-dependent manner, indicating a role in modulation of channel gating.

In addition, the quintuple mutant was analyzed which exhibits amino acid exchanges from serine to alanine at all five potential phosphorylation sites (S768, S814, S835, S892 and S928). This mutant was also functional showing similar maximally induced current densities as the wildtype (Figure 7A,B,I,J). Interestingly, the quintuple mutant exhibited distinct effects on current kinetics compared to the quadruple mutant. Notably, OptoBI-1-induced fast inactivation was significantly slower compared to the wildtype and the quadruple mutant (Figure 7G), while other OptoBI-1-induced kinetic parameters remained unchanged compared to the wildtype. In contrast, OptoDArG-induced activation and deactivation kinetics were significantly slower compared to the wildtype (Figure 7L,M). However, OptoDArG-induced fast and slow inactivation kinetics were unaffected (Figure 7O,P).

NSC analysis of OptoBI-1- and OptoDArG-induced current–voltage relationships revealed significant differences in the NSC curve progressions, particularly in the quadruple and quintuple mutants (Supplemental Figure S11). Interestingly, even the OptoDArG-induced currents of the double mutant displayed slight deviations in NSC curve progression at negative potentials.

### 2.8. PKC Phosphorylation or Dephosphorylation Alters the Current Kinetics

**Figure 8:**
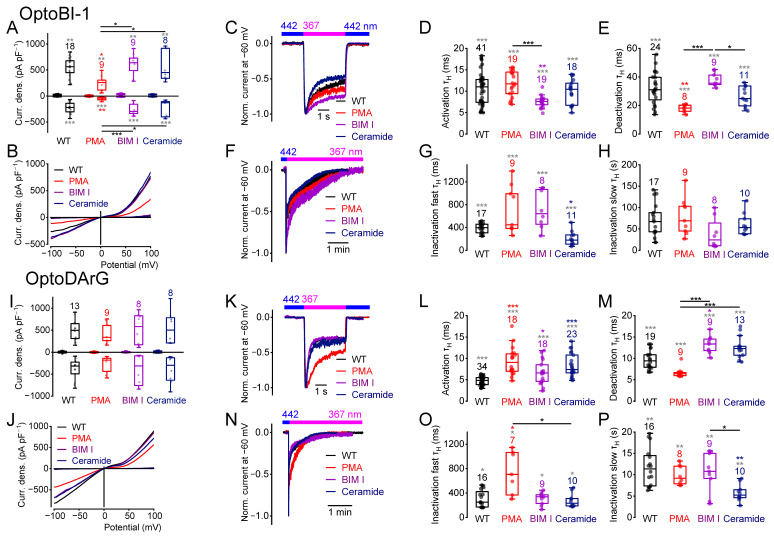
PKC phosphorylation and dephosphorylation alter the current kinetics. Electrophysiological whole-cell measurements of HEK293T cells overexpressing TRPC6 incubated with the PKC activator PMA (1 µM) or with the PKC inhibitors BIM I (1 µM) or ceramide (2 µM) for 20 min at room temperature in the presence of 10 µM OptoBI-1 (**A**–**H**) or 30 µM OptoDArG (**I**–**P**). (**A**,**I**) Summaries of current densities (‘Curr. dens.’) at potentials of ±100 mV evoked by light. First small boxplots represent current densities in the presence of blue light, which establishes *trans*-configuration, and second boxplots represent maximal current densities in the presence of UV light, which establishes *cis*-configuration. Significant differences were observed between *cis*-OptoBI-1-induced current densities in the presence of PMA compared to the wildtype. (**B**,**J**) Representative current-density–voltage relations induced by illumination with UV light. (**C**,**F**,**K**,**N**) Representative normalized current time courses of inward currents at constant holding potential of -60 mV during photoswitching from blue light (blue bar) to UV light (magenta bar) (**F**,**N**) and back to blue light (blue bar) (**C**,**K**). (**D**,**E**,**L**,**M**) Summaries of half-life time constants (τ_H_) of the activation (**D**,**L**) and deactivation (**E**,**M**) kinetics. (**G**,**H**,**O**,**P**) Summaries of half-life time constants (τ_H_) of the fast (**G**,**O**) and slow (**H**,**P**) inactivation kinetics. (**D**,**E**,**G**,**H**,**L**,**M**,**O**,**P**) Data are displayed as boxplots and interquartile ranges. Numbers over boxplots indicate number of measured cells. Gray asterisks indicate overall differences among all groups identified by the Kruskal–Wallis test, whereas colored and black asterisks indicate pairwise differences revealed by Dunn’s post hoc analysis (colored vs. wild type; black between the groups connected by horizontal lines). * *p* < 0.05; ** *p* < 0.01; *** *p* < 0.001.

To evaluate whether activation or inhibition of the PKC influenced current densities and/or current kinetics, we incubated HEK293T cells overexpressing wildtype TRPC6 with the potent PKC activator PMA or with the PKC inhibitors bisindolylmaleimide I (BIM I) or ceramide (N-acetyl-L-erythro-sphingosine) for 20 min at room temperature. Incubation, with PMA (1 µM) to induce PKC phosphorylation, resulted in reduced maximal *cis*-OptoBI-1-induced, but not *cis*-OptoDArG-induced current densities (Figure 8A,B,I,J) compared to the wildtype. The *cis*-OptoBI-1-induced current densities of PMA treated cells were significantly smaller compared to the BIM I- and ceramide-treated cells. Furthermore, PKC activation caused faster OptoBI-1-induced deactivation kinetics compared to the wildtype (Figure 8E). However, OptoDArG-induced activation and fast inactivation kinetics were significantly slower compared to the wildtype (Figure 8L,O).

Incubation with BIM I (1 µM) or ceramide (2 µM) had no significant effect on the maximally induced current densities compared to the wildtype (Figure 8A,B,I,J). However, OptoBI-1-induced current densities in the presence of BIM I or ceramide were significantly higher than those in PMA-treated cells (Figure 8A). BIM I and ceramide elicited distinct effects on the current kinetics. BIM I, but not ceramide, which inhibits PKC isoforms such as PKC-ε [62] and PKC-α [63] but activates other PKC isoforms such as PKC-ζ [64,65], significantly enhanced OptoBI-1-induced activation kinetics (Figure 8D). While BIM I had no effect, ceramide provoked enhanced fast inactivation kinetics in the presence of OptoBI-1 compared to the wildtype (Figure 8G). OptoDArG-induced current kinetics were also affected. Both BIM I and ceramide caused significantly slower activation kinetics (Figure 8L). BIM I, but not ceramide, resulted in slower deactivation kinetics compared to the wildtype (Figure 8M). However, the deactivation kinetics in the presence of both BIM I and ceramide were significantly slower compared to the treatment with PMA (Figure 8M). Furthermore, OptoDArG-induced slow inactivation kinetics were significantly faster after incubation with ceramide, but not with BIM I (Figure 8P). Altogether, these findings suggest substance-specific effects of PKC activation or inhibition on TRPC6 current kinetics. Pharmacological PKC modulation altered the current kinetics, suggesting that the TRPC6 channel might exhibit PKC phosphorylation sites that are constitutively phosphorylated and others that are not phosphorylated under physiological conditions in the overexpression system.

In addition, calculation of the NSC revealed significant differences in OptoBI-1- and OptoDArG-induced NSC curve progressions at positive potentials (Supplemental Figure S12) after incubation with PMA. NSC curve progressions of the OptoBI-1 induced currents in the presence of BIM I or ceramide were also significantly different over a wide potential range (Supplemental Figure S12A) suggesting that pharmacological PKC inhibition influences the channel gating behavior. Altogether, PKC modulation altered the TRPC6 current kinetics in an activator-specific manner, thereby changing the channel gating.

### 2.9. The Quadruple Mutant Incubated with Ceramide Behaves Like the Quintuple Mutant

**Figure 9:**
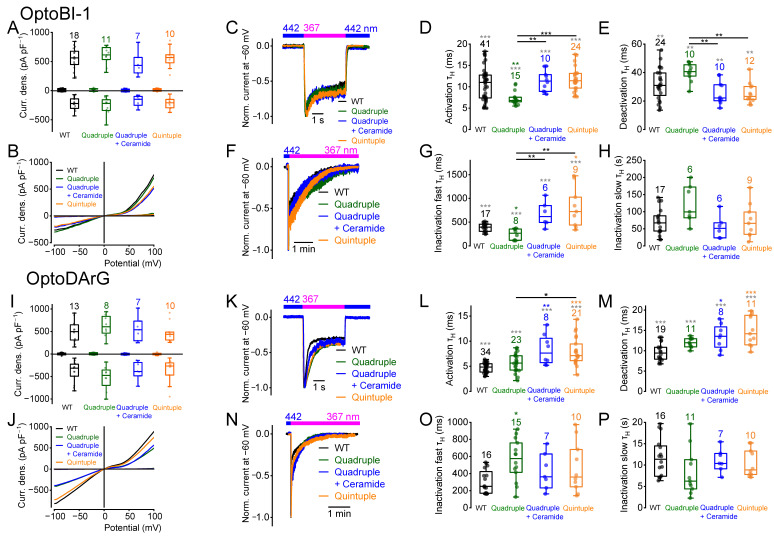
The quadruple mutant incubated with ceramide behaves like the quintuple mutant. Electrophysiological whole-cell measurements of HEK293T cells overexpressing TRPC6 or the TRPC6 quadruple (S768A, S814A, S835A and S892A) mutant, the quadruple mutant incubated with the PKC inhibitor ceramide (2 µM for 20 min at room temperature), or the quintuple (S768A, S814A, S835A, S892A and S928A) mutant in the presence of 10 µM OptoBI-1 (**A**–**H**) or 30 µM OptoDArG (**I**–**P**). (**A**,**I**) Summaries of current densities (‘Curr. dens.’) at potentials of ±100 mV evoked by light. First small boxplots represent current densities in the presence of blue light, which establishes *trans*-configuration, and second boxplots represent maximal current densities in the presence of UV light, which establishes *cis*-configuration. (**A**) No significant differences were observed between *trans-*OptoBI-1-induced or *cis*-OptoBI-1-induced current densities of the mutant channels compared to the wildtype. (**I**) *trans*-OptoDArG- or *cis*-OptoDArG-induced current densities of the mutant channels were not significantly different compared to the wildtype. (**B**, **J**) Representative current-density–voltage relations induced by illumination with UV light. (**C**,**F**,**K**,**N**) Representative normalized current time courses of inward currents at constant holding potential of -60 mV during photoswitching from blue light (blue bar) to UV light (magenta bar) (**F**,**N**) and back to blue light (blue bar) (**C**,**K**). (**D**,**E**,**L**,**M**) Summaries of half-life time constants (τ_H_) of the activation (**D**,**L**) and deactivation (**E**,**M**) kinetics. (**G**,**H**,**O**,**P**) Summaries of half-life time constants (τ_H_) of the fast (**G**,**O**) and slow (**H**,**P**) inactivation kinetics. (**D**,**E**,**G**,**H**,**L**,**M**,**O**,**P**) Data are displayed as boxplots and interquartile ranges. Numbers over boxplots indicate number of measured cells. Gray asterisks indicate overall differences among all groups identified by the Kruskal–Wallis test, whereas colored and black asterisks indicate pairwise differences revealed by Dunn’s post hoc analysis (colored vs. wild type; black between the groups connected by horizontal lines). * *p* < 0.05; ** *p* < 0.01; *** *p* < 0.001.

As mentioned above, both quadruple and quintuple mutants showed individual differences in their current kinetics (see Figure 7). Therefore, we next determined whether additional incubation of the quadruple mutant with ceramide to possibly prevent phosphorylation of the remaining C-terminal phosphorylation site S928 results in similar current kinetics than the quintuple mutant. Incubation of the quadruple mutant with ceramide did not change the maximally induced current density amplitudes (Figure 9A,B,I,J). Furthermore, similar OptoBI-1- and OptoDArG-induced current kinetics, as seen in the quintuple mutant, were observed (Figure 9D–P), suggesting that an additional PKC phosphorylation at serine 928 might be responsible for the observed differences in the current kinetics between the quadruple and the quintuple mutant. The NSC curve progression of the quadruple mutant significantly differed over a wide potential range in the presence of OptoBI-1 compared to the quintuple mutant (Supplemental Figure S13A). However, the NSC curve progression of the quadruple mutant in the presence of ceramide did not differ from that of the quintuple mutant, even when activated with OptoDArG (Supplemental Figure S13B), suggesting that ceramide might result in dephosphorylation of serine 928. However, the NSC curve progressions of the quadruple and quintuple mutants activated with OptoDArG did not differ from each other anyway, suggesting that in the presence of OptoDArG, the quadruple mutant might already be dephosphorylated at position serine 928 or that other regulatory mechanisms occur in the presence of DAG derivatives.

**Figure 10:**
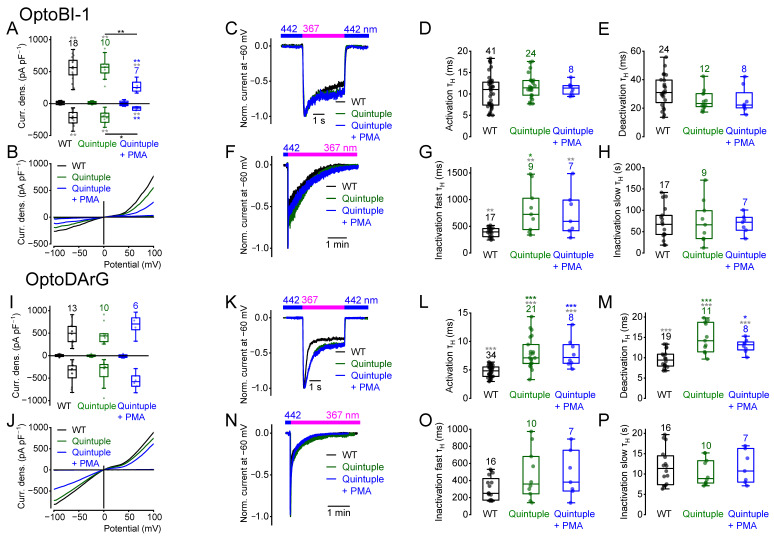
Additional PKC activation did not alter the current kinetics of the quintuple mutant in electrophysiological whole-cell measurements of HEK293T cells overexpressing TRPC6, the TRPC6 quintuple (S768A, S814A, S835A, S892A, and S928A) mutant, or the quintuple mutant incubated with the PKC activator PMA (1 µM for 20 min at room temperature) in the presence of 10 µM OptoBI-1 (**A**–**H**) or 30 µM OptoDArG (**I**–**P**). (**A**,**I**) Summaries of current densities (‘Curr. dens.’) at potentials of ±100 mV evoked by light. First small boxplots represent current densities in the presence of blue light, which establishes *trans*-configuration, and second boxplots represent maximal current densities in the presence of UV light, which establishes *cis*-configuration. Significant differences between *cis*-OptoBI-1-induced current densities of the quintuple mutant incubated with PMA compared to the wildtype channel were observed. (**B**,**J**) Representative current-density–voltage relations induced by illumination with UV light. (**C**,**F**,**K**,**N**) Representative normalized current time courses of inward currents at constant holding potential of -60 mV during photoswitching from blue light (blue bar) to UV light (magenta bar) (**F**,**N**) and back to blue light (blue bar) (**C**,**K**). (**D**,**E**,**L**,**M**) Summaries of half-life time constants (τ_H_) of the activation (**D**,**L**) and deactivation (**E**,**M**) kinetics. (**G**,**H**,**O**,**P**) Summaries of half-life time constants (τ_H_) of the fast (**G**,**O**) and slow (**H**,**P**) inactivation kinetics. (**D**,**E**,**G**,**H**,**L**,**M**,**O**,**P**) Data are displayed as boxplots and interquartile ranges. Numbers over boxplots indicate number of measured cells. Gray asterisks indicate overall differences among all groups identified by the Kruskal–Wallis test, whereas colored and black asterisks indicate pairwise differences revealed by Dunn’s post hoc analysis (colored vs. wild type; black between the groups connected by horizontal lines). * *p* < 0.05; ** *p* < 0.01; *** *p* < 0.001.

In addition, the quintuple mutant was incubated with the PKC activator PMA to eventually promote additional PKC phosphorylation. Interestingly, *cis*-OptoBI-1-, but not *cis*-OptoDArG-induced maximal current densities were significantly reduced compared to the wildtype and to the quintuple mutant in the absence of PMA (Figure 10A,B,I,J), indicating that PMA might elicit additional phosphorylation at other potential PKC phosphorylation sites, thereby reducing channel activity or eventually the membrane expression of the quadruple mutant in the presence of OptoBI-1 but not of OptoDArG. However, analysis of the current kinetics revealed that additional incubation of the quintuple mutant with PMA resulted in similar current kinetics as the untreated quintuple mutant (Figure 10) suggesting that PKC activation—although it influenced the maximal current density amplitudes—had no effect on the current kinetics of the quintuple mutant. These findings might suggest that the five C-terminal phosphorylation sites fully accounted for the observed changes in current kinetics, thus making the involvement of other phosphorylation sites unlikely. The corresponding NSC curve progressions of the quintuple mutant incubated with PMA in the presence of OptoBI-1 revealed only marginal differences at positive potentials of around +50 mV (Supplemental Figure S13) indicating that although the *cis*-OptoBI-1-induced current density amplitudes were reduced, the channel gating was unaffected by incubation with PMA.

### 2.10. Dephosphorylation of Endogenously Expressed TRPC6 Channels Results in Increased Current Density Amplitudes and in Slower Current Kinetics

**Figure 11:**
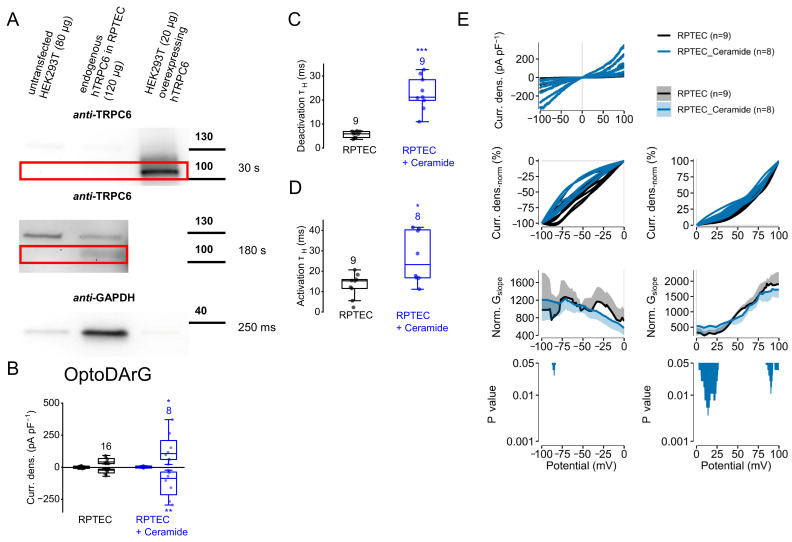
PKC inhibition alters current kinetics and normalized slope conductance of endogenously expressed TRPC6 channels. (**A**) Western blot analysis of HEK293T cells, HEK293T cells overexpressing human TRPC6, and of human renal proximal tubule endothelial cells (RPTEC) endogenously expressing low amounts of TRPC6. The red box shows human TRPC6 expression at 106 kDa. The amount of protein used, and the different exposure times are indicated on the top or, respectively, to the right of the images. (**B**–**E**) Electrophysiological whole-cell measurements of RPTEC and of RPTEC incubated with ceramide (2 µM for 20 min at room temperature) in the presence of 30 µM OptoDArG. (**B**) Summary of current densities (‘Curr. dens.’) at potentials of ±100 mV evoked by light. First small boxplots represent current densities in the presence of blue light, which establishes *trans*-configuration, and second boxplots represent maximal current densities in the presence of UV light, which establishes *cis*-configuration. Significant differences between *cis*-OptoDArG-induced current densities of RPTEC in the presence or absence of ceramide were observed (* *p* < 0.05, ** *p* < 0.01; Mann–Whitney U test). (**C**,**D**) Summaries of half-life time constants (τ_H_) of the activation (**C**) and deactivation (**D**) kinetics (* *p* < 0.05, *** *p* < 0.001; Mann–Whitney U test). (**B**–**D**) Data are displayed as boxplots and interquartile ranges. Numbers over boxplots indicate number of measured cells. (**E**) Current-density–voltage relations (‘Curr. dens.’) of *cis*-OptoDArG-induced currents in the presence or absence of ceramide are displayed (above). The current-density–voltage relations were separately smoothed and normalized (‘Curr. dens_norm_ (%)’) at negative and positive potentials. The calculated normalized slope conductance (NSC) (‘Norm. G_slope_’) is displayed as mean ± SD. *p* values are calculated using Mann–Whitney U-test.

To assess the physiological relevance of TRPC6 phosphorylation, we next examined endogenously expressed TRPC6 channels in human proximal tubule endothelial cells (RPTEC). A low endogenous expression of TRPC6 in RPTEC was confirmed by Western blot (Figure 11A). In renal tubule cells, TRPC6 channels might be involved in several pathophysiological states, such as ischemia–reperfusion injury [66], as well as in tumorigenesis and progression of renal cell carcinoma, which predominately originates from the proximal tubule [67]. We used OptoDArG for TRPC6 activation, since DAG serves as an endogenous activator of TRPC6 channels. To analyze the effect of PKC inhibition on endogenously expressed TRPC6 currents, we incubated RPTEC with ceramide, since ceramide had caused more pronounced effects on the OptoDArG-induced current kinetics than BIM I (see Figure 8). Analyzing *cis*-OptoDArG-induced current densities, we found that ceramide caused significantly higher current density amplitudes (Figure 11B) indicating that PKC inhibition enhances the channel activity of endogenously expressed TRPC6, which was not observed when analyzing heterologously overexpressed TRPC6 channels in HEK293T cells (see Figure 8I). Furthermore, the current density amplitudes obtained from endogenously expressed channels in RPTEC were much smaller than in the overexpression system. However, OptoDArG-induced activation and deactivation kinetics were both significantly slower in the presence of ceramide (Figure 11C,D) which was in line with the findings in the overexpression system (Figure 8L,M) suggesting that endogenously expressed TRPC6 channels in RPTEC might possess permanent PKC phosphorylation sites that accelerate their activation and deactivation kinetics. Analysis of the NSC curve progression showed significant differences between ceramide-treated and untreated cells, mainly at positive potentials (Figure 11E). In contrast, the NSC curve progressions in the presence and absence of ceramide of TRPC6, which was heterologously overexpressed in HEK293T cells, were unchanged (Supplemental Figure S13B), suggesting differences between the endogenous and the heterologous expression system. But since it was reported that renal proximal tubule cells might also endogenously express TRPC3 channels, heteromeric TRPC3/6 channel complexes with different current characteristics were possible [9,68]. However, our findings suggest that the PKC phosphorylation status of endogenously expressed TRPC6 channels influences channel activity and channel-gating behavior, suggesting that PKC phosphorylation is important for the regulation and fine-tuning of the channel state.

## 3. Discussion

Our results highlight an interplay between PKC-dependent signaling, site-directed mutagenesis, and activator-specific alterations in TRPC6 current kinetics. However, since we did not biochemically confirm phosphorylation at individual residues, these findings should be interpreted as consistent with, rather than definitive proof of evidence of phosphorylation-dependent modulation. Accordingly, the term phosphorylation is used in a functional sense, referring to PKC-dependent modulation inferred from mutagenesis and pharmacological interventions. The pharmacological modulators used in this study (PMA, BIM I, and ceramide) exert complex and context-dependent effects, indicating that their impact on TRPC6 activity cannot be attributed solely to PKC modulation. To substantiate the link between TRPC6 current kinetics and PKC phosphorylation, future biochemical analyses such as phospho-mapping or mass spectrometry will be required to verify site-specific phosphorylation and to establish causal relationships.

However, in line with previous work, in this study we confirm that photopharmacology allows precise and reproducible characterization of TRP channel activation, deactivation, and inactivation kinetics via whole-cell current recordings [51,52]. This photopharmacological approach is sufficiently sensitive to detect subtle alterations in current kinetics. However, it does not directly prove PKC-mediated phosphorylation. Accurate kinetic analysis, however, requires high-intensity, fast-switching light sources and photoswitchable activators at saturating concentrations [51,52]. Beyond temporal precision, photopharmaceuticals also permit spatial control over channel activity, offering the potential of reducing systemic side effects in vivo [69]. A limitation of many azobenzene-based switches is their requirement for UV light for *cis*-isomerization, which is phototoxic and poorly penetrates tissue. New developments such as the red-light-switchable compound dfdc-OptoBI-1 (∼620 nm) improve tissue compatibility [60], but slow activation kinetics currently limit its use in experiments requiring rapid switching. We therefore employed the structurally distinct, photoswitchable TRPC6 activators OptoDArG [21] and OptoBI-1 [53], which elicit different gating mechanisms, as indicated by their kinetic profiles [51] and current–voltage analyses, including NSC calculations [61]. These complementary tools provide a robust basis for extended biophysical characterization of TRPC6.

Until now, a detailed investigation of how C-terminal phosphorylation modulates TRPC6 kinetics was lacking. Insights from TRPC5 indicate that amino acid exchange from threonine to alanine in the PDZ-binding motif to prevent PKC phosphorylation can slow current inactivation and alter DAG sensitivity [16,26] via dissociation of the scaffold proteins NHERF1/2. Since TRPC6 lacks this motif, analogous mechanisms were speculative. Although PKC-mediated phosphorylation has been reported for multiple TRPC isoforms, the kinetic consequences remain unclear.

TRPC6 possesses four putative C-terminal PKC phosphorylation sites (S768, S835, S892, and S928 in mouse and S769, S836, S893, and T929 in human TRPC6). Earlier studies suggested that DAG can directly activate TRPC6 channels through binding within the pore region and that this activation is independent of PKC [14]. In addition, it was reported that PKC activation with PMA suppresses receptor-operated, but not OAG-induced, TRPC6 channel activation [34] in the heterologous overexpression system and in podocytes [70], pointing to a complex interplay between PKC signaling and TRPC6 function, particularly in pathological states such as proteinuria. In our study, we found that PMA reduced the *cis*-OptoBI-1-induced current densities, whereas the *cis*-OptoDArG-induced responses remained unaffected. Consistent with previous findings [34], our results confirm that after PMA treatment, the maximal current amplitudes evoked by DAG derivatives remain unchanged. However, we show that PKC activators and inhibitors exert compound-specific effects on TRPC6 current kinetics—an insight that was not achievable with conventional approaches, which further highlights the advantage of photopharmacological tools. PMA treatment reduced the deactivation of TRPC6 in response to OptoBI-1 and had distinct effects on OptoBI-1- and OptoDArG-induced activation and inactivation. PKC inhibitors such as BIM I and ceramide also yielded distinct effects: ceramide altered fast inactivation with OptoBI-1, while in OptoDArG-stimulated cells, fast inactivation was unaffected compared to the wildtype. These results underline the compound-specific nature of PKC modulation and indicate that PKC activation fine-tunes the temporal characteristics of TRPC6 activity. The divergent effects observed with the PKC inhibitors BIM I and ceramide likely reflect their distinct isoform selectivity and additional signaling effects. Ceramide is known to inhibit certain conventional and novel PKC isoforms while activating atypical PKC-ζ [62,63,64,65]. Moreover, ceramide can activate protein phosphatase 2A (PP2A) [71] and inhibit Akt [72] and ERK signaling [73], indicating broader regulatory actions beyond PKC inhibition. Therefore, some of the observed effects on TRPC6 current kinetics may result from indirect or off-target mechanisms rather than from PKC inhibition.

The observed activator-specific effects may result from different mechanisms of action of OptoDArG and OptoBI-1, including distinct modulation of the PKC activity. An important methodological consideration is that prolonged pre-incubation with *trans*-OptoDArG (~20 min) may partly inhibit PKC, as this compound suppresses basal TRPC3 currents [51]. Since kinase activation can occur within seconds to minutes [74,75,76], this pre-incubation could alter PKC activity, and thus the TRPC6 phosphorylation status, and thereby contribute to differences in current amplitudes and current kinetics between OptoBI-1 and OptoDArG stimulation. However, to validate these compound-specific effects and to precisely map the phosphorylation status of serine and threonine residues of TRPC6 under basal conditions and upon exposure to PKC modulators, additional studies are required. Furthermore, the detailed gating mechanism of TRPC channels remains incompletely understood, as no 3D structure of TRPC channels in the open state is currently available and little is known about the molecular basis of activation, deactivation and inactivation. Therefore, kinetic analyses cannot yet be directly linked to specific gating transitions, which will require further structural and mechanistic studies.

The observed regulatory mechanisms extend beyond overexpression systems. In renal proximal tubule epithelial cells, which endogenously express TRPC6 (and possibly TRPC3), PKC inhibition slowed OptoDArG-induced kinetics and increased current density amplitudes, suggesting physiological relevance in renal pathophysiology, including ischemia–reperfusion injury [66] and renal cancer [67]. Similar PKC-dependent regulation has been reported for TRPC1, affecting endothelial permeability [46,48] and insulin secretion [50], indicating that PKC phosphorylation serves as a broader regulatory principle for TRPC channels. Further studies are required to elucidate the physiological and pathophysiological relevance of this PKC-dependent TRPC6 modulation in more detail. In proximal tubule epithelial cells, only ceramide was tested as a PKC inhibitor; future experiments using additional modulators and different cell types, together with a more precise characterization of phosphorylation sites and gating mechanisms, will help clarify how PKC influences TRPC6 function. Notably, in many cases PKC modulation and amino acid substitutions did not alter maximal current amplitudes but only affected channel kinetics and changes in the NSC slope progression, indicating that changes in channel kinetics can modulate the channel-gating behavior [51]. The physiological consequences of these effects remain to be investigated. In addition, the relatively small number of recorded cells per condition represents a limitation. However, the effects were generally consistent across experiments.

Mutagenesis of the four C-terminal PKC sites to alanine (to prevent phosphorylation) or aspartate/glutamate (to approximate the negative charge introduced by constitutive phosphorylation) revealed site- and activator-specific effects on kinetics and on current amplitudes. In some cases, significant differences were observed between alanine and glutamate or aspartate mutants, for example, in the activation (Figure 1L, Figure 2D and Figure 4D), the deactivation (Figure 2E,M, Figure 4E, Figure 5E,M and Figure 6E), and the slow inactivation kinetics (Figure 2H) as well as in the maximally induced current density amplitudes (Figure 4I) which supports the view that phosphorylation or dephosphorylation at these sites may modulate TRPC6 gating. Importantly, substitutions to aspartate or glutamate did not always replicate the effects of true phosphorylation, highlighting the structural and electrostatic complexity of phosphate recognition. However, our findings suggest a complex interplay between PKC modulation and site-specific amino acid substitutions in shaping TRPC6 function.

PKC phosphorylation of S768 in rat TRPC6 reduces channel activity [35]. In our analysis, substitution to alanine or to aspartate and glutamate at S768 did not affect maximal current amplitudes for either activator, but did alter specific kinetic parameters, supporting the value of detailed kinetic analysis. S768 is highly conserved across TRPC isoforms (corresponds to S669 in mouse TRPC1, S962 in mouse TRPC2, S774 in mouse TRPC3, S662 in mouse TRPC4, S666 in mouse TRPC5, and S714 in mouse TRPC7), and analogous residues in TRPC3 and TRPC7 similarly influence activity [45], with additional effects on cytoskeletal architecture in myofibroblasts [45]. Comparable roles for homologous sites in TRPC1, TRPC2, TRPC4, and TRPC5 remain unexplored.

The conserved threonine T573 in human TRPC3 (T629 in mouse TRPC6) resides in the S4–S5 linker near TM5 and is a potential PKC target. While phosphorylation at this site in TRPC3 did not alter current amplitudes, it promoted TRPC3-calcineurin complex formation and NFAT activation [44]. Whether homologous modifications influence TRPC6 kinetics needs further investigation.

Serine 814 in mouse TRPC6 (S815 in human) is constitutively phosphorylated in human disease tissues [37,38,39,40,41,42] yet is not conserved in other TRPC isoforms. Consistent with previous studies [37], we found that amino acid exchange to alanine had no effect on TRPC6 channel activity, determined as maximally induced current density amplitudes. Amino acid exchange to aspartate or glutamate also did not alter the channel activity. However, kinetic changes were observed in an activator-dependent manner. For instance, OptoBI-1 changed the activation, deactivation, and both fast and slow inactivation kinetics, while OptoDArG only affected deactivation kinetics. These results reinforce that kinetic parameters can reveal regulatory effects invisible in amplitude measurements and may be relevant for disease-associated fine-tuning of channel function.

Similarly, the non-conserved S835 did not affect maximal amplitudes but altered specific current kinetics, suggesting a modulatory role in gating. By contrast, S892, which is conserved among several TRPC channels, affected both amplitudes and kinetics in an activator-dependent manner. Although structural data place S892 within the coiled-coil domain and potentially inaccessible to phosphorylation, mutation-induced effects on kinetics suggest altered conformational dynamics.

Comparison of quadruple and quintuple alanine mutants showed that S928 is essential to reproduce the kinetic phenotype seen with PKC inhibition by ceramide. PMA had no effect on the current kinetics of the quintuple mutant, indicating that additional PKC sites outside the C-terminus are unlikely to contribute to the observed effects. Located near the C-terminal end, S928 may be functionally analogous to T972 in TRPC5, known to affect inactivation kinetics [26]. To probe the structural relevance of this region, we introduced mutations S928A and S928G, as well as truncations at residues 928 and 929. Both amino acid exchanges altered the channel kinetics in distinct ways but had no effect on the maximally induced current densities. However, deletion of the final three residues (including S928) resulted in enhanced, rather than decelerated, slow inactivation in the presence of both activators, indicating that S928 does not share functional equivalence with T972 in TRPC5. Furthermore, the mutant S928* mostly mimicked the effects of S928G, whereas truncation of the last two residues had no impact. These findings confirm a role of C-terminal PKC phosphorylation sites in fine-tuning channel gating.

Other non-canonical PKC phosphorylations, or phosphorylations induced by other protein kinases, might also affect channel function and possibly current kinetics. N-terminal phosphorylations by protein kinase A (PKA), protein kinase G (PKG), and Rho-associated coiled-coil containing protein kinase 1 (Rock1) also modulate TRPC6 activity. For instance, PKA phosphorylation at S28 and T69 reduces TRPC6 activity [77,78,79], while PKG and ROCK1 may target additional residues [80]. Whether C-terminal phosphorylation also affects protein–protein interactions or conformational states remains unclear. Further structural–functional studies will be necessary to define the full spectrum of phosphorylation-dependent regulation [81].

TRPC inactivation mechanisms remain poorly understood. Current inactivation is an intrinsic feature of the channel that might be influenced, e.g., by phosphorylations [26,36]. Here, we demonstrate that amino acid exchanges at potential C-terminal phosphorylation sites and pharmacological PKC modulation can influence both the fast and slow components of inactivation, which serve as empirical descriptors derived from bi-exponential fitting. It may be speculated that these components correspond to mechanistically distinct gating transitions underlying fast and slow phases of inactivation. Further studies employing state-dependent mutagenesis, single-channel electrophysiology, or time-resolved structural approaches will be required to elucidate the mechanistic basis of these kinetic components. In many cases, changes in fast inactivation were linked to altered NSC curve progression, suggesting that this parameter could serve as a functional marker of gating modulation. This was particularly evident in the mutant S768A, S768D, S768E, S814A, S892E, and S928A (in the presence of OptoBI-1), S892D and S892E (in the presence of OptoDArG), in the quadruple (in the presence of OptoBI-1 and OptoDArG), and the quintuple mutant (in the presence of OptoBI-1), and after the incubation of wildtype TRPC6 with ceramide (in the presence of OptoBI-1) or PMA (in the presence of OptoDArG). Altogether, this association was observed in multiple mutants and pharmacological conditions, though not universally, indicating contributions from additional mechanisms. However, the underlying molecular mechanism remains unclear and should be further investigated in future studies.

In summary, our findings establish photopharmacology as a sensitive and versatile approach for dissecting TRPC6 kinetics and identifying phosphorylation-dependent regulatory mechanisms. Although direct phosphorylation was not demonstrated, our mutagenesis and pharmacological data are consistent with a C-terminal PKC-dependent modulation of TRPC6 gating in a site- and activator-specific manner, producing subtle but functionally relevant changes in current kinetics without necessarily affecting peak current amplitudes. These phosphorylation events may shape TRPC6 function in physiological and pathological contexts, particularly in the kidney. A detailed kinetic perspective thus adds a new dimension to TRPC6 functional analysis and opens avenues for targeted modulation in disease.

## 4. Material and Methods

### 4.1. Data Availability

All data reported in the paper and any additional information required to reanalyze the data reported in this paper is available from the lead contact upon request.

### 4.2. Cell Lines Used in the Study

In this study, we used human embryonic kidney (HEK293T) cells (from Leibniz-Institute DSMZ, Braunschweig, Germany, T293, DSMZ no. ACC 635) and human renal proximal tubule epithelial (RPTEC/TERT1) cells (from ATCC, Manassas, VA, USA; Cat. No. CRL-4031).

### 4.3. Materials

Poly-L-Lysine (Cat. No. P-1524), Bovine serum albumin (BSA; Cat. No. A7030), Bisindolylmaleimide I (BIM I; Cat. No. 203290), and Phorbol-12-myristat-13-acetat (PMA; Cat. No. P8139) were purchased from Sigma-Aldrich (Taufkirchen, Germany). OptoDArG was purchased from Aobious (Gloucester, MA, USA; Cat. No. AOB31427), OptoBI-1 was purchased from Bio-Techne (Minneapolis, MN, USA; Cat. No. 7013). Ceramide (N-Acetyl-D-erythro-sphingosine; Cat. No. A15377) was purchased from Hölzel Diagnostika Handels GmbH (Köln, Germany). OptoDArG was dissolved in anhydrous DMSO to 50 mM, OptoBI-1 was dissolved in anhydrous DMSO to 10 mM, BIM I, and PMA was dissolved in anhydrous DMSO to 1 mM, ceramide was dissolved in anhydrous DMSO to 20 mM. OptoDArG stock solutions were stored in aliquots at −20 °C for maximal 4 weeks, all other stock solutions were stored in aliquots at −20 °C for maximal 1 year.

### 4.4. Molecular Biology and Mutagenesis

The primers for site-directed mutagenesis (SDM) were automatically designed using the NEBaseChanger (nebasechanger.neb.com) and purchased from Sigma-Aldrich (Specifications: Desalt, in solution (water), 100 µM). Primer sequences and annealing temperatures are listed in Table 1.

The plasmid used in this study was a pIRES2-EGFP expression vector (Clontech, Palo Alto, CA, USA) containing mTRPC6 cDNA (NM_013838). Amino acid substitutions in mTRPC6 were introduced via SDM using the Q5 Site-directed Mutagenesis KIT (New England Biolabs GmbH, Frankfurt am Main, Germany; Cat. No. E0552S) following the manufacturer’s protocol. For SDM, polymerase chain reaction (PCR) was performed using FastGene Ultra Cycler Gradient (Nippon Genetics, Düren, Germany; Cat. No. FG-TC01). Plasmid DNA was extracted using the HiYield Plasmid Mini Kit (Süd-Laborbedarf GmbH, Gauting, Germany; Cat. No. 30 HYPD100) according to the manufacturer’s instructions. The presence of the correct mutation was confirmed via Sanger sequencing, performed by Eurofins Genomics (Luxembourg).

### 4.5. Cell Culture and Transfection

HEK293T cells were kept in Earl’s MEM (Sigma-Aldrich) with 100 units/mL penicillin and 100 μg/mL streptomycin supplemented with 10% (vol/vol) FCS (Gibco, Life Technologies, Carlsbad, CA, USA). RPTEC were kept in DMEM:F12 Medium (ATCC; Cat. No. 30-2006) combined with hTERT Immortalized RPTEC Growth Kit (ATCC; Cat. No. ACS-4007). All cells were held at 37 °C in a humified atmosphere with 5% CO_2_. HEK293T cells were transfected with 2 µg cDNA coding for mouse TRPC6 (NM_013838) using Genejuice reagent (Sigma-Aldrich) according to the manufacturer’s instructions. The cDNA was in pIRES2-EGFP expression vector (Clontech, Palo Alto, CA, USA). Transfected HEK293T cells were trypsinated for maximal 3 s, resuspended in culture medium and subsequently seeded onto poly-L-Lysine-coated glass cover slips (diameter 30 mm, thickness 1, Karl Hecht, Sondheim, Germany) 1 h prior to the patch-clamp measurements.

### 4.6. Light Stimulation

For light stimulation, two LEDs from Thorlabs (M365LP1 with a peak wavelength of 367 nm and M450LP2 with a peak wavelength of 442 nm; Bergkirchen, Germany) with optical band pass filters from AHF (360/23 BrightLine HC and 433/24 BrightLine HC; Tübingen, Germany) were used, which were connected over dichroic beamsplitters from AHF (HC 376 and HC 458, respectively) and which elicited effective light with wavelengths between 346 and 371 nm and between 418 and 447 nm. An Olympus IX70 microscope with dichroic beamsplitter (H 488 LPXR superflat Vers. 2) combined with the emission filter (525/50 BrightLine HC) from AHF and the 40× oil UV-transmissive apochromatic objective (UApo N 340; Evident, Hamburg, Germany) were used. The LEDs were operated by a self-made control unit with IC-HG30 laser switches mounted on EVAL HG1D evaluation boards from iC-Haus (Bodenheim, Germany) connected to the microcontroller board Arduino Mega 2560 (Arduino SA, Chiasso, Switzerland). A self-written MATLAB app (R2024a; MathWorks Inc.; Natick, MA, USA) served as a user-interface driving the microcontroller through serial communication [51]. The effective time of switching between the two LEDs was less than 5 µs. The LEDs operated with intensity levels of an 8-bit sigmoidal scale.

### 4.7. Patch-Clamp Recordings

Patch-clamp recordings were performed as previously described [51]. OptoBI-1 and OptoDArG stock solutions were heated to 40 °C for 10 min for short time thermal relaxation and subsequently diluted in standard bath solution containing 140 mM NaCl, 5 mM CsCl, 1 mM MgCl_2_, 2 mM CaCl_2_, 10 mM glucose, and 10 mM HEPES (pH 7.4 with NaOH resulting in an osmolarity of 295–302 mOsm·kg^−1^) to a final concentration of 10 µM for OptoBI-1 and 30 µM for OptoDArG. The solutions were illuminated with blue light prior to application to the cells. For some indicated measurements, PMA, BIM I, or ceramide were additionally added to the standard bath solution resulting in final concentrations of 1 µM for PMA and BIM I and 2 µM for ceramide. Conventional whole-cell patch-clamp recordings were carried out at room temperature (23 °C) 48 h after transfection. The mTRPC6 over-expressing HEK293T or RPTEC seeded on coverslips were incubated with 30 µM OptoDArG solution for 20 to 30 min at room temperature prior to the measurements. OptoBI-1 (10 µM) required no preliminary incubation. PMA (1 µM) or BIM I (1 µM) and ceramide (2 µM) were also incubated for 20 min at room temperature prior to the patch-clamp measurements.

OptoDArG, OptoBI-1, PMA, and BIM I were used in their maximally effective concentrations. Higher concentrations of ceramide compromised cell membrane stability, rendering measurements at concentrations of ceramide above 2 µM unfeasible. The standard pipette solution contained 120 mM CsCl, 9.4 mM NaCl, 0.2 mM Na_3_-GTP, 1 mM MgCl_2_, 3.949 mM CaCl_2_, 10 mM BAPTA (100 nM free Ca^2+^), and 10 mM HEPES (pH 7.2 with CsOH), resulting in an osmolality of 294 mOsm kg^−1^. The liquid junction potential of +4.0 mV was calculated by JPCalcWin 1.01 (University of New South Wales, Sydney, Australia) and was corrected before the measurements. Data were collected with an EPC10 patch-clamp amplifier (HEKA Elektronik, Lambrecht, Germany) using the Patchmaster software version v2x90.5. Transfected cells were selected by illumination with light of wavelength 442 nm to detect green fluorescent protein EGFP.

Cells were allowed to equilibrate with the pipette solution for about 2 to 4 min after break-in, depending on the pipette resistance, before recordings were started. For the determination of current-density–voltage relationships and normalized slope conductances (NSC), repetitive voltage upramps from −100 to +100 mV were applied. Current-density analysis and current-density–time courses were determined at holding potentials of +100 and −100 mV. For determination of the current density and of current-density–voltage relations, a stimulation protocol was used with a frequency of 50 Hz starting with a holding potential of −100 V for 7 ms, followed by a voltage upramp from −100 to +100 V for 10 ms and a holding potential of +100 mV for 3 ms. Hereby, data was acquired at a frequency of 5 kHz after filtering at 2.5 kHz. For the stimulation protocol with voltage upramps, alternating light stimulation (blue–UV–blue) was applied two to three times to obtain maximally induced steady-state current densities. Kinetics of channel activation, deactivation, and inactivation were obtained at a holding potential of −60 mV with a sampling frequency of 4 kHz. Three consecutive light stimulations per cell were conducted to determine activation and deactivation kinetics. Activation and deactivation kinetics were evaluated separately for each stimulation. As kinetic speed did not correlate with stimulation number, the fastest kinetics per cell were selected for analysis. To determine inactivation kinetics, UV light was applied until the current returned to the values of the baseline current before application of UV light. The protocol for current inactivation was applied only once. Immediately after applying the inactivation protocol, the currents could not be reactivated upon subsequent stimulation. Patch pipettes were made of borosilicate glass from Science Products (Hofheim, Germany; Cat. No. GB150TF-8P) and had resistances of 1.8 to 3.2 MΩ. We did not apply any leak current subtraction; data sets exhibiting detectable leak currents were excluded, and only high-quality recordings were analyzed.

### 4.8. Normalized Slope Conductance

The NSC were calculated using current–voltage relations that were selected at maximal activator-induced current amplitudes in the presence of UV light. The calculation was performed as described in detail by Hermann et al. [61]. Briefly, current-density–voltage relations were smoothed with a cubic spline fit and normalized. Subsequently, the NSC was calculated from the separately smoothed and normalized inward and outward current densities at potential ranges from −100 to 0 mV and from 0 mV to +100 mV. *p* values were calculated and displayed separately over the whole potential range using an unpaired Mann–Whitney-U test.

### 4.9. Fit Routine

The determination of the half-life time constants for the current kinetics were performed as previously described [51]. In short, the half-life time constants of the activation, deactivation, and fast and slow inactivation (τ_H_) current kinetics were first normalized. Than the time was shifted to zero for fitting using MATLAB R2024a. For τ_H_ of the activation kinetics, the median of the current before activation was set to zero and about 30% of the maximal activated current was set to +1 to exclude eventually interfering inactivation. For τ_H_ of the deactivation kinetics, the median of the current before deactivation was set to +1 and the median of the fully deactivated current was set to zero. For τ_H_ of inactivation, the peak current was set to +1 and the median of the steady-state current after inactivation was set to zero. Activation and deactivation were fitted by a mono-exponential function (Formulas (1) and (2)), inactivation was fitted by a bi-exponential function (Formula (3)) (see Supplemental Figure S3) since a bi-exponential fit consistently provided a superior description of the current decay (higher r^2^) compared to a mono-exponential fit, and both kinetic components were reproducibly observed, including across all tested mutants. Fit optimization was performed with a quadratic error function calculating the summed square of residuals (SSE, Formulas (4) and (5)) using the fminsearch function and the options described in Table 2.

(1) $$f_{activation}(t)=a*e^{\frac{ln2*t}{\tau_{H}}}+c$$

Formula (1): Mono-exponential fit function *f_activation_* dependent on time (*t*), initial quantity (*a*), time constant (*τ_H_*), and offset (*c*)

(2) $$f_{deactivation}(t)=a*e^{-\frac{ln2*t}{\tau_{H}}}+c$$

Formula (2): Mono-exponential fit function *f_deactivation_* dependent on time (*t*), initial quantity (*a*), time constant (*τ_H_*), and offset (*c*)

(3) $$f_{inactivation}(t)=a_{1}*e^{-\frac{ln2*t}{\tau_{H1}}}+a_{2}*e^{-\frac{ln2*t}{\tau_{H2}}}+c$$

Formula (3): Bi-exponential fit function *f_inactivation_* dependent on time (*t*), initial quantities (*a*_1_; *a*_2_), time constants (*τ_H_*_1_, *τ_H_*_2_), and offset (*c*)

(4) $$SSE=\Sigma_{t}{\left[y_{t}-f\left(a,c,\;\tau_{H},t\right)\right]}^{2}$$

Formula (4): Quadratic error function for mono-exponential fits using the summed square of residuals (SSE) with *y_t_* being the current at time *t* and *f*(*a*, *c*, *τ_H_*, *t*) the result either of Formula (1) or Formula (2) at time *t*

(5) $$SSE=\Sigma_{t}{\left[y_{t}-f\left(a_{1},a_{2},c,\tau_{H1},\tau_{H2},t\right)\right]}^{2}$$

Formula (5): Quadratic error function for bi-exponential fits using the summed square of residuals (SSE) with *y_t_* being the current at time *t* and *f*(*a*, *c*, *τ_H_*_1_, *τ_H_*_2_, *t*) the result of Formula (3) at time *t*.

### 4.10. Western Blot

For cell lysis, HEK293T and RPTEC were cultivated in a 150 mm TC-treated cell culture dish, seeded to a density of 90% confluency. For hTRPC6 overexpressing HEK293T cells, transfection was carried out using GeneJuice reagent (Sigma-Aldrich; Cat. No.: 70967-5) and 2 µg of cDNA coding for hTRPC6 for 24 h. Medium was aspirated, and the cells were washed twice in 10 mL ice-cold PBS. Next, 1 mL of ice-cold RIPA buffer (Sigma-Aldrich; Cat. No. R0278) was applied. The cells were then scraped off the plate using a cell-scraper, collected in a tube and gently shaken at 4 °C for 30 min. Afterwards, cell debris was sedimented at 14,000× *g* for 30 min at 4 °C. The supernatant was collected and a bicinchoninic acid assay (BCA assay) was performed using the Pierce^TM^ BCA Protein Assay Kit (Thermo Fisher Scientific Inc., Waltham, MA, USA; Cat. No. 23227) according to the manufacturer’s protocol in double determination.

For SDS-PAGE and Western blot, protein samples were prepared and diluted in 4X Laemmli buffer and incubated at 95 °C for 10 min. Protein samples were then loaded onto 4–15% Mini-PROTEAN TGX^TM^ Precast Protein Gels (Bio-Rad Laboratories GmbH, Feldkirchen, Germany; Cat. No. 4561084). SDS-PAGE was performed at 100 V for 75 min. Blotting onto PVC membrane was performed using wet blot approach with Bio-Rad Laboratories Blotting System at 100 V for 1 h.

All subsequent washing steps were performed with 0.1% Tween20 in TBS buffer (TBS-T). Blocking was performed with 5% milk powder in TBS-T. Afterwards, rabbit anti-TRPC6 antibody (Proteintech, Planegg-Martinsried, Germany; Cat. No.: 18236-1-AP) was diluted 1:1000 in TBS-T containing 5% BSA and the blot was incubated for 12–18 h at 4 °C. Next, the secondary antibody (goat anti-rabbit-HRP antibody, Cell Signaling Technology, Leiden, The Netherlands; Cat. No.: 7074S, 1:1000) in 5% BSA in TBS-T-solution was applied for 90 min at 23 °C. Images were acquired with ChemiDoc following incubation with Clarity ECL Soution (Bio-Rad; Cat. No. 170506). Afterwards, the membrane was stripped with mild stripping buffer (0.15% Glycin, 0.1% SDS, 1% Tween20 adjusted to pH 2.2 with HCl) for 20 min at 23 °C. Then it was blocked with 5% milk powder in TBS-T for 1 h. Next, the mouse anti-GAPDH-HRP antibody 1:2000 (from Proteintech; Cat. No. HRP-6004) was incubated for 12–18 h at 4 °C. Images were acquired with ChemiDoc after incubation with Clarity ECL Solution.

### 4.11. Quantification and Statistical Analysis

Statistical analyses were conducted in R 4.2.3 and in Origin 2025 (OriginLabs, Northhampton, MA, USA). No statistical methods were used to predetermine sample size. The *p* values were calculated by using an unpaired Mann–Whitney U or Kruskal–Wallis test with Dunn’s multiple comparison post hoc analysis. A *p* value less than 0.05 was considered significant for all analysis. * *p* < 0.05; ** *p* < 0.01; *** *p* < 0.001. Boxplots display the median and the interquartile range. Values of half-life time constants (*τ_H_*) are indicated as mean ± standard deviation (SD). The statistical tests used for statistical analysis can be found in the figure legends.

## Figures and Tables

**Table 1:** Primer sequences and annealing temperature for SDM.

Mutation	Primer Name	Primer Sequence (5′-3′)	Annealing Temperature [°C]
S768A	mTRPC6_S768A_f	TCTTGTACCAgccCCAAAATCCTTGCTTTATC	61
	mTRPC6_S768A_r	TTGAAGGGGACAGGAAGTG	
S768D	mTRPC6_S768D_f	TCTTGTACCAgacCCAAAATCCTTGCTTTATC	61
	mTRPC6_S768D_r	TTGAAGGGGACAGGAAGTG	
S768E	mTRPC6_S768E_f	TCTTGTACCAgaaCCAAAATCCTTGCTTTATC	61
	mTRPC6_S768E_r	TTGAAGGGGACAGGAAGTG	
S814A	mTRPC6_S814A_f	AATTTCAGGAgccCACGAAGACCTTTC	57
	mTRPC6_S814A_r	CCAAATTTCTTTTCTTCATTTCTC	
S814D	mTRPC6_S814D_f	AATTTCAGGAgacCACGAAGACCTTTC	57
	mTRPC6_S814D_r	CCAAATTTCTTTTCTTCATTTCTC	
S814E	mTRPC6_S814E_f	AATTTCAGGAgaaCACGAAGACCTTTC	57
	mTRPC6_S814E_r	CCAAATTTCTTTTCTTCATTTCTC	
S835A	mTRPC6_S835A_F	CAAACAATCAgccACAAGGAGCTCAGAAG	60
	mTRPC6_S835A_R	TTGTGTGCCAACTGATTTTTG	
S835D	mTRPC6_S835D_f	CAAACAATCAgacACAAGGAGCTCAGAAG	59
	mTRPC6_S835D_r	TTGTGTGCCAACTGATTTTTG	
S835E	mTRPC6_S835E_f	CAAACAATCAgaaACAAGGAGCTCAGAAG	59
	mTRPC6_S835E_r	TTGTGTGCCAACTGATTTTTG	
S892A	mTRPC6_S892A_f	AGACATCTCAgccCTCCGTTATGAAC	56
	mTRPC6_S892A_r	TGCTTAATTTCCTTCAATTC	
S892D	mTRPC6_S892D_f	AGACATCTCAgacCTCCGTTATGAAC	56
	mTRPC6_S892D_r	TGCTTAATTTCCTTCAATTC	
S892E	mTRPC6_S892E_f	AGACATCTCAgaaCTCCGTTATGAAC	56
	mTRPC6_S892E_r	TGCTTAATTTCCTTCAATTC	
S928A	mTRPC6_S928A_f	GCTGGAGGAAgccCGCAGATAGC	62
	mTRPC6_S928A_r	TTTGGCTCTAACGACAGTC	
S928D	mTRPC6_S928D_f	GCTGGAGGAAgacCGCAGATAGC	63
	mTRPC6_S928D_r	TTTGGCTCTAACGACAGTC	
S928E	mTRPC6_S928E_f	GCTGGAGGAAgaaCGCAGATAGC	58
	mTRPC6_S928E_r	TTTGGCTCTAACGACAGTC	
S928G	mTRPC6_S928G_f	GCTGGAGGAAggcCGCAGATAGC	67
	mTRPC6_S928G_r	TTTGGCTCTAACGACAGTCTCTC	
S928*	mTRPC6_S928Stopp_f	GCTGGAGGAAtaaCGCAGATAGCC	60
	mTRPC6_S928Stopp_r	TTTGGCTCTAACGACAGTC	
R929*	mTRPC6_R929Stopp_f	GGAGGAAAGCtaaAGATAGCCCTAC	59
	mTRPC6_R929Stopp_r	AGCTTTGGCTCTAACGAC	

**Table 2:** Parameters and values for fit routine.

Parameter	Value
MaxFunEvals	5000
MaxIter	10,000
TolX	1 × 10^−10^
TolFun	1 × 10^−6^
Initial values activation	*a* = 0.01; *τ_H_* = 1; *c* = 0.01
Initial values deactivation	*a* = 0.7; *τ_H_* = 7; *c* = 0.1
Initial values inactivation	*a*_1_ = 1; *τ_H_*_1_ = 0.02; *c* = 0.1, *a*_2_ = 1, *τ_H_*_2_ = 3.8

## Data Availability

The original contributions presented in this study are included in the article/Supplementary Materials. Further inquiries can be directed to the corresponding author.

## Supplementary Materials

The following supporting information can be downloaded at: https://www.mdpi.com/article/10.3390/ijms262311482/s1.
