# Supplementary material for: Impact of C-Terminal PKC Phosphorylation on TRPC6 Current Kinetics
Source: Int J Mol Sci. 2025 Nov 27;26(23):11482. doi: 10.3390/ijms262311482 (PMC12692586; doi:10.3390/ijms262311482)
Supplement: Supplementary file 1 [file ijms-26-11482-s001.zip › ijms-3845109-supplementary.pdf]

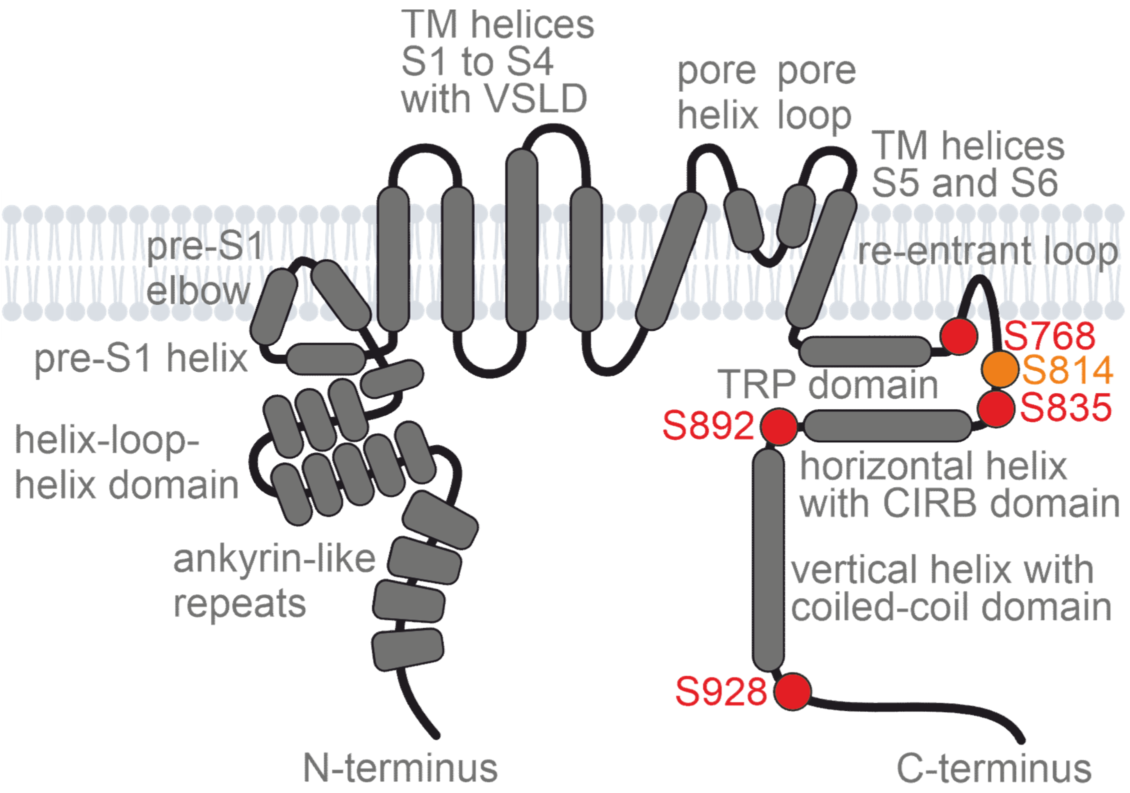


**Supplemental Figure 1: Schematic representation of a TRPC6 monomer**. Important structural features are displayed. The four C-terminal PKC phosphorylation sites (S768, S835, S892 and S928) are displayed in red and the phosphorylation site S814 is displayed in orange.

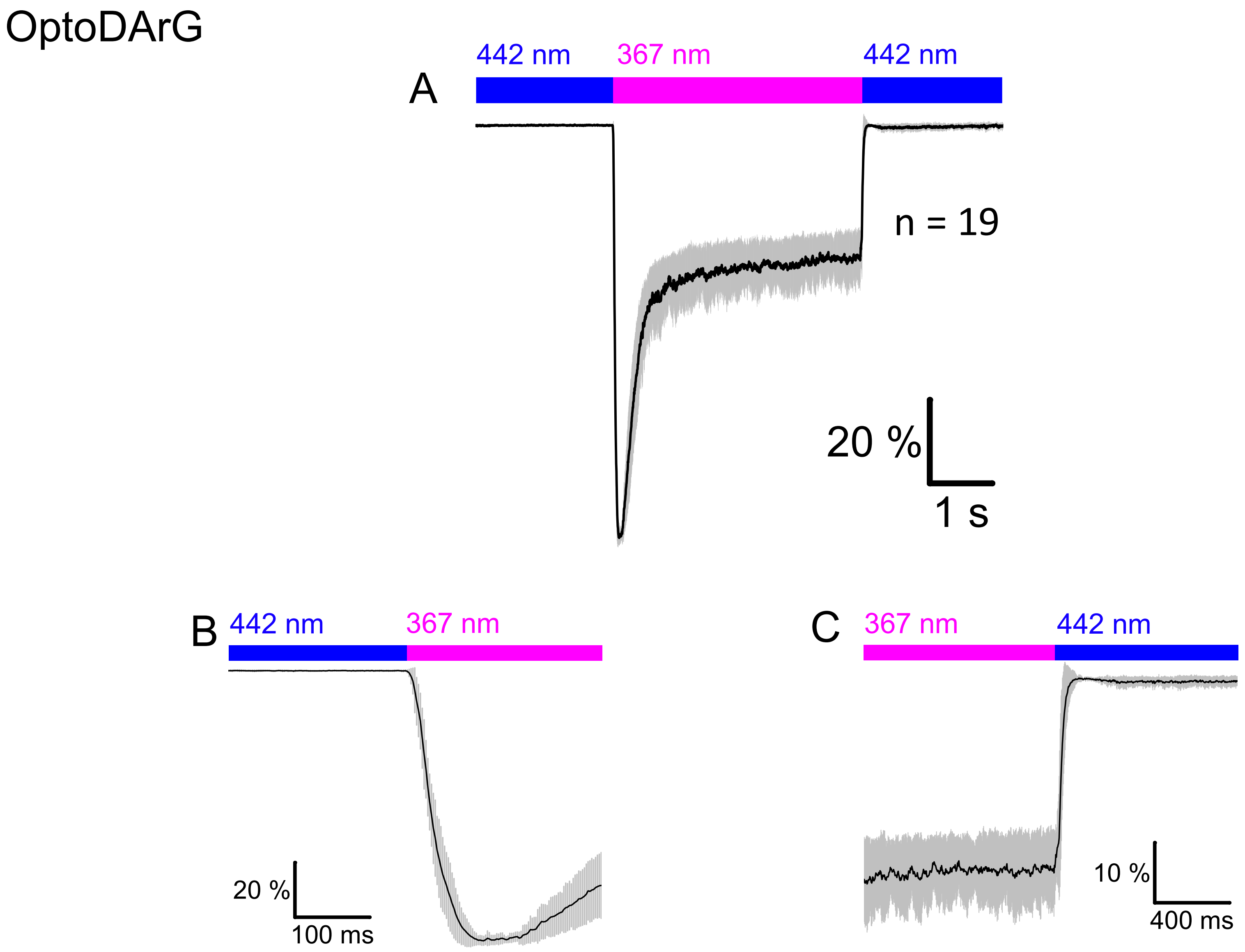


**Supplemental Figure 2: Averaged traces of normalized current time courses.** Electrophysiological whole-cell measurements of HEK293T cells overexpressing TRPC6 in the presence of 30 µM OptoDArG. (**A**-**C**) Averaged normalized current time courses of inward currents at constant holding potential of -60 mV during photoswitching from blue light (blue bar) to UV light (magenta bar) and back to blue light (blue bar) displayed as median ± SD. **Related to Figure 1**.

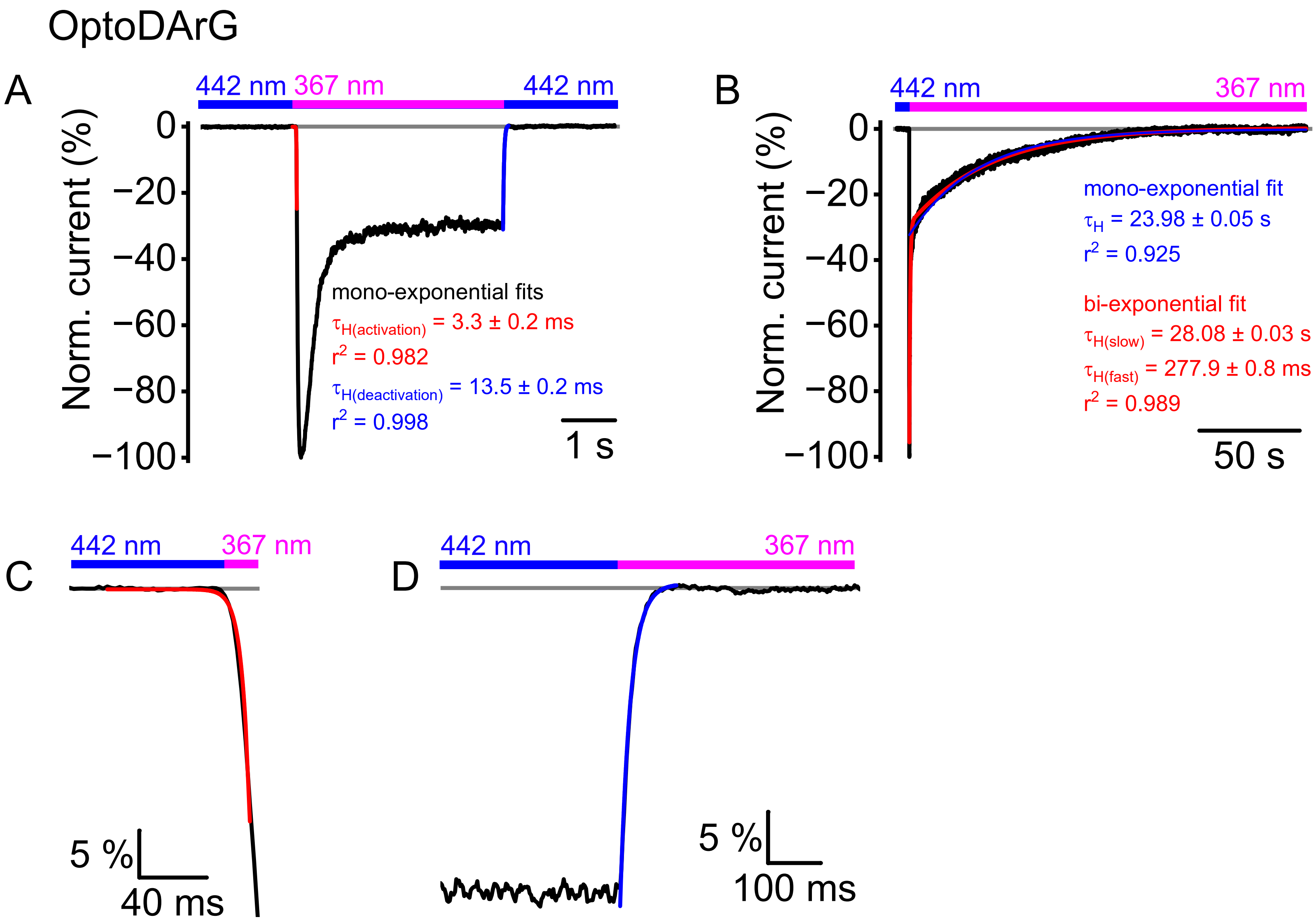


**Supplemental Figure 3: Fit Routine for calculation of the half-life constant $\tau_H$ of the activation, deactivation and inactivation kinetics.** Whole-cell measurements of TRPC6 overexpressing HEK293T cells in the presence of 30 µM OptoDArG. (**A** and **B**) Representative normalized current time courses of inward currents at constant holding potential of -60 mV during photoswitching from blue light (blue bar) to UV light (magenta bar) (**A** and **B**) and back to blue light (blue bar) (**B**). (**A**) Mono-exponential fits for the activation and deactivation kinetics demonstrating a good agreement between model and data ($R^2$=0.982 or 0.009, respectively).and calculated half-life constant $\tau_H$ for activation and deactivation kinetics. (**B**) Representative mono-expononential and bi-exponential fits for the inactivation kinetics. Bi-exponential fit provides a better description of the data ($r^2$ = 0,989) than the mono-exponential fit ($r^2$ = 0,925). Bi-exponential fit provides evidence for a fast and a slow component of the inactivation. Calculated half-life constants $\tau_H$ for the inactivation kinetics are displayed. (**C** and **D**) Zoom-in views into the current time course (**A**) showing mono-exponential fits for activation (**C**) and deactivation (**D**) kinetics. **Related to Figure 1.**

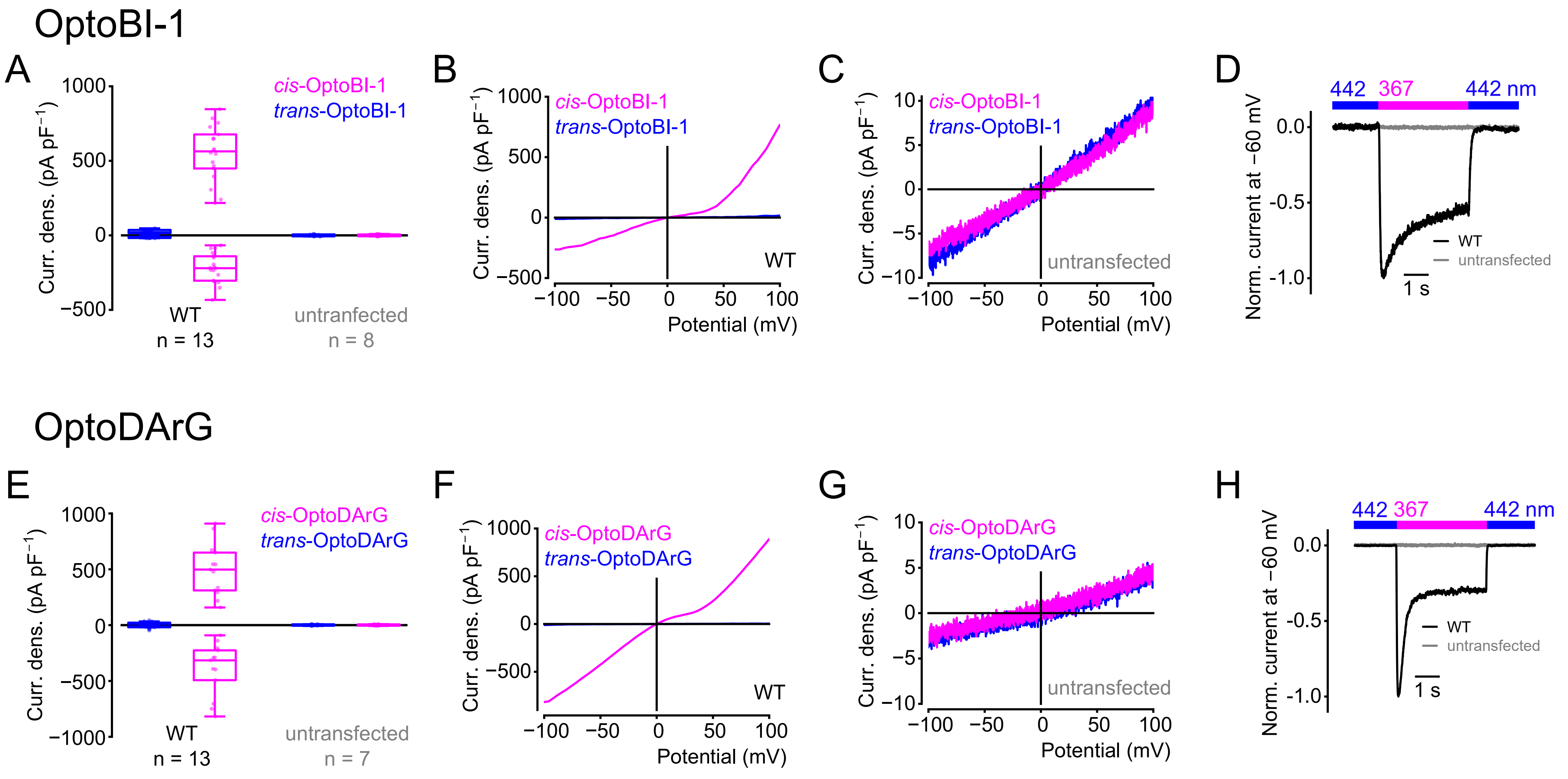


**Supplemental Figure 4: Untransfected HEK293T cells show no current changes during phososwitching.** Whole-cell measurements of untransfected and TRPC6 overexpressing HEK293T cells in the presence of 10 µM OptoBI-1 (**A**-**D**) or 30 µM OptoDArG (**E**-**H**). (**A** and **E**) Summaries of current densities ('Curr. dens.') at potentials of ±100 mV evoked by light. First small boxplots represent current densities in the presence of blue light which establishes *trans*-configuration and second boxplots represents maximal current densities in the presence of UV light which establishes *cis*-configuration. (**B**, **C**, **F**, **G**) Representative current density-voltage relations induced by illumination with blue and UV light of TRPC6 overexpressing HEK192T cells (**B** and **F**) and of untransfected HEK293T cells (**C** and **G**). (**D** and **H**) Representative normalized time courses of inward currents at constant holding potential of -60 mV during photoswitching from blue light (blue bar) to UV light (magenta bar) and back to blue light (blue bar). Numbers over boxplots indicate number of measured cells**. Related to Figure 1.**

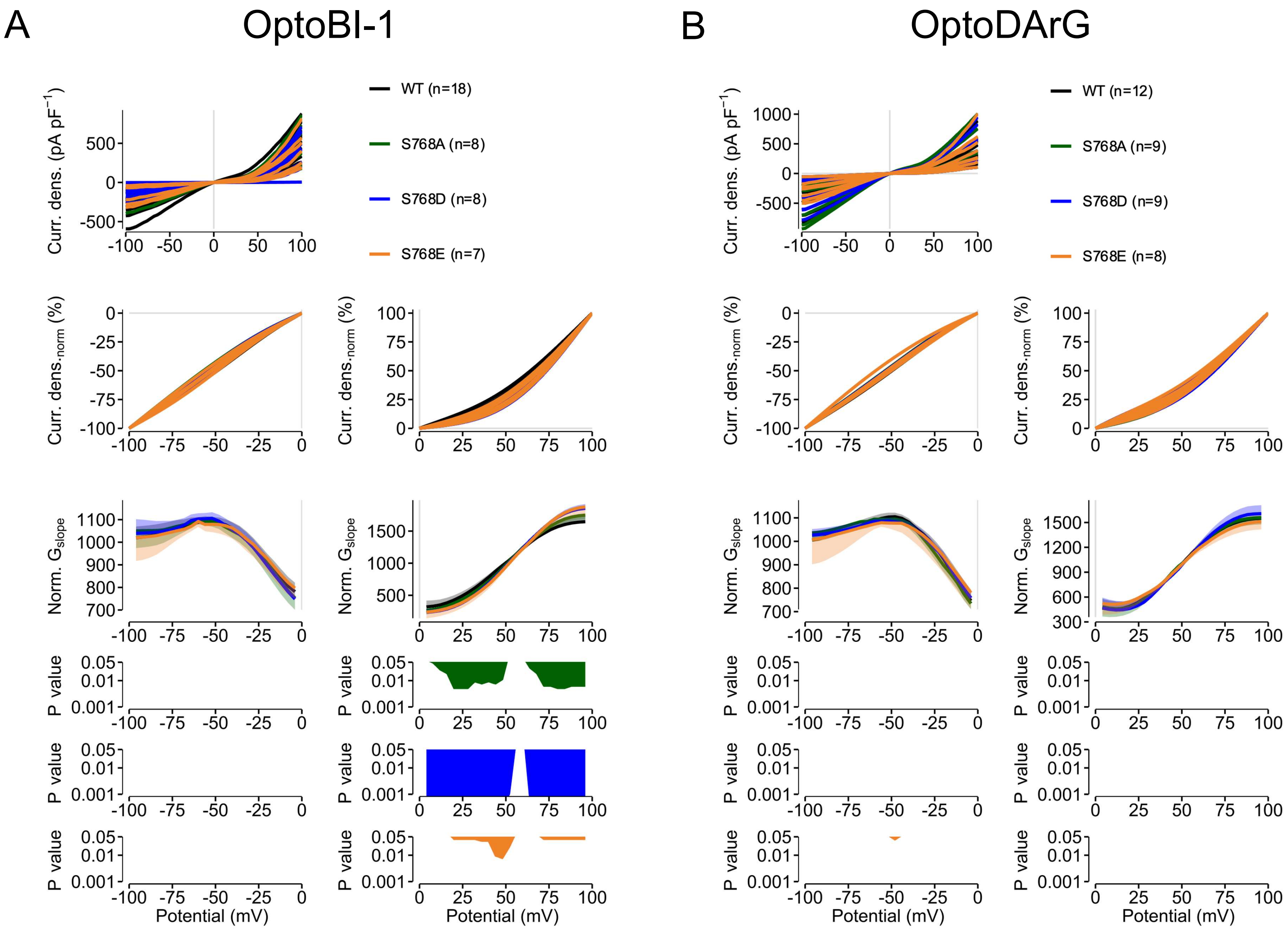


**Supplemental Figure 5: Normalized slope conductance of TRPC6 mutants with amino acid exchanges at S768.** Whole-cell measurements of TRPC6 and indicated TRPC6 mutants overexpressing HEK293T cells. (**A**, **B**) Current density-voltage relations ('Curr. dens.') induced by *cis*-OptoBI-1 (**A**) or *cis*-OptoDArG (**B**) are displayed above. The current density-voltage relations were separately smoothed and normalized ('Curr. $dens_{norm}$ (%)') at positive and negative potentials. The calculated normalized slope conductance (NSC) ('Norm. $G_{slope}$') is displayed as mean ± SD. P values are calculated using Mann-Whitney U test compared to wildtype TRPC6. **Related to Figure 1.**

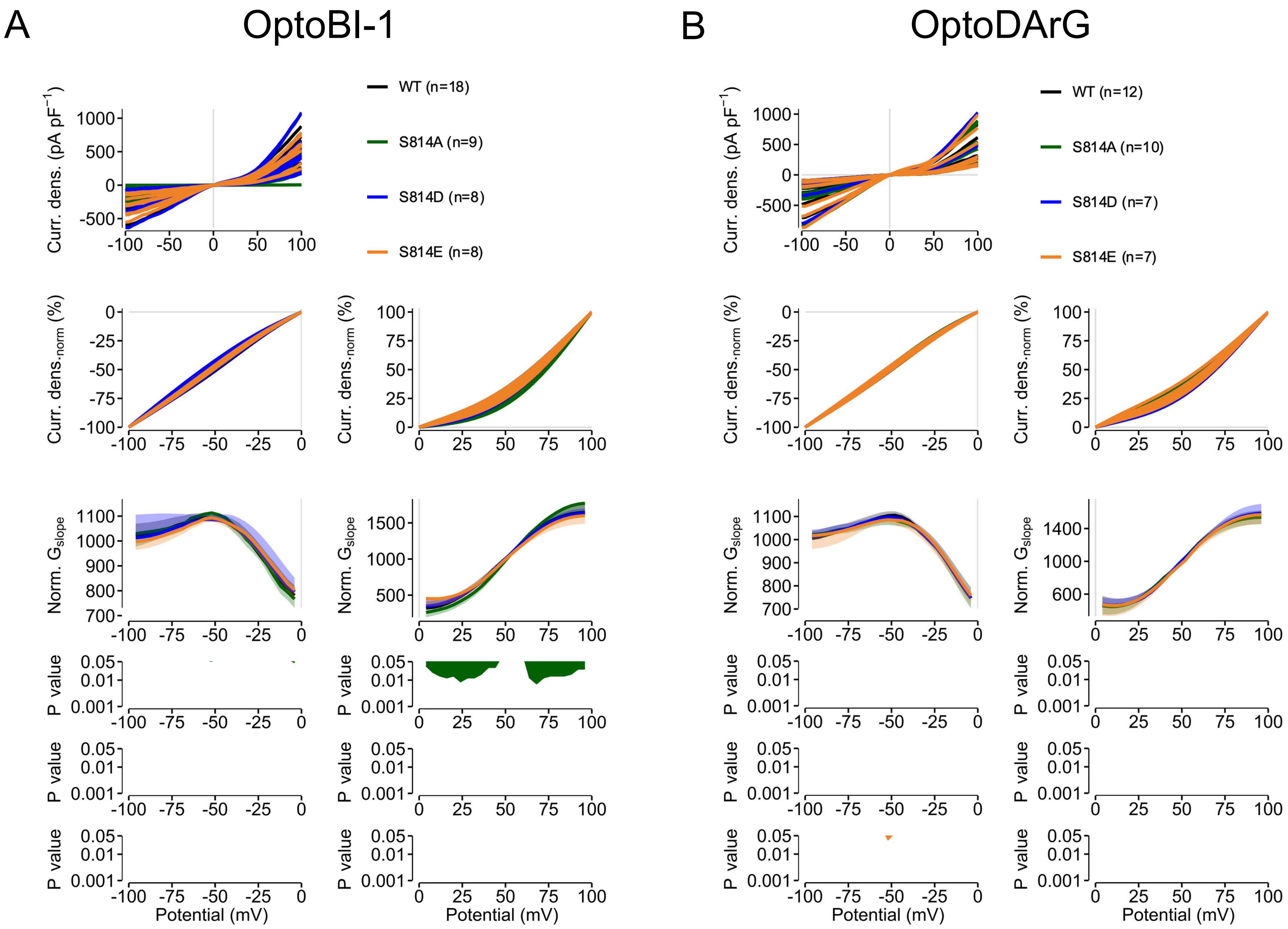


**Supplemental Figure 6: Normalized slope conductance of TRPC6 mutants with amino acid exchanges at S814.** Whole-cell measurements of TRPC6 and indicated TRPC6 mutants overexpressing HEK293T cells. (**A**, **B**) Current density-voltage relations ('Curr. dens.') induced by *cis*-OptoBI-1 (**A**) or *cis*-OptoDArG (**B**) are displayed above. The current density-voltage relations were separately smoothed and normalized ('Curr. $dens_{norm}$ (%)') at positive and negative potentials. The calculated normalized slope conductance (NSC) ('Norm. $G_{slope}$') is displayed as mean ± SD. P values are calculated using Mann-Whitney U test. test compared to wildtype TRPC6 **Related to Figure 2.**

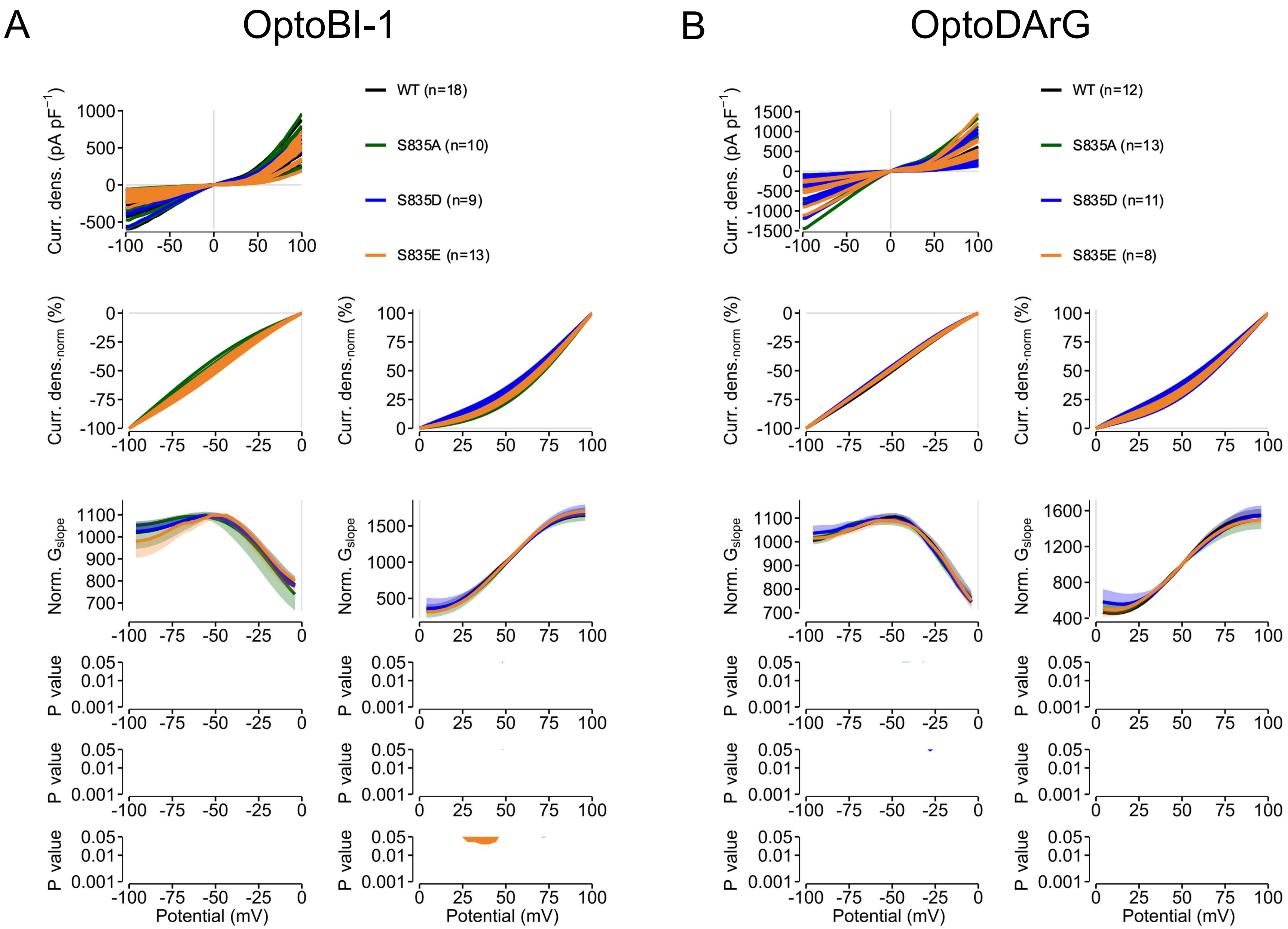


**Supplemental Figure 7: Normalized slope conductance of the TRPC6 mutants with amino acid exchanges at S835.** Whole-cell measurements of TRPC6 and indicated TRPC6 mutants overexpressing HEK293T cells. (**A**, **B**) Current density-voltage relations ('Curr. dens.') induced by *cis*-OptoBI-1 (**A**) or *cis*-OptoDArG (**B**) are displayed above. The current density-voltage relations were separately smoothed and normalized ('Curr. $dens_{norm}$ (%)') at positive and negative potentials. The calculated normalized slope conductance (NSC) ('Norm. $G_{slope}$') is displayed as mean ± SD. P values are calculated using Mann-Whitney U test test compared to wildtype TRPC6. **Related to Figure 3.**

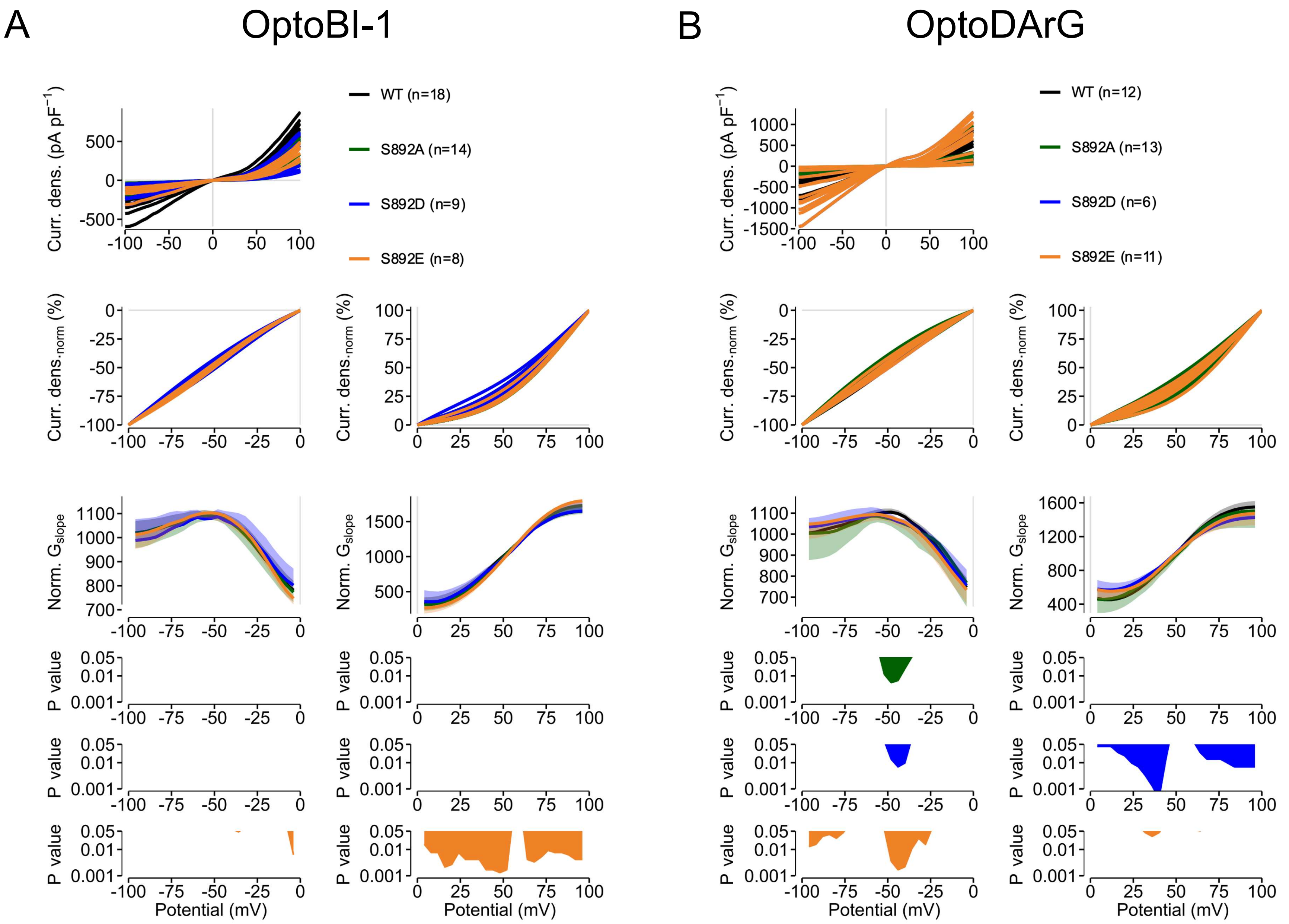


**Supplemental Figure 8: Normalized slope conductance of TRPC6 mutants with amino acid exchanges at S892.** Whole-cell measurements of TRPC6 and indicated TRPC6 mutants overexpressing HEK293T cells. (**A**, **B**) Current density-voltage relations ('Curr. dens.') induced by *cis*-OptoBI-1 (**A**) or *cis*-OptoDArG (**B**) are displayed above. The current density-voltage relations were separately smoothed and normalized ('Curr. $dens_{norm}$ (%)') at positive and negative potentials. The calculated normalized slope conductance (NSC) ('Norm. $G_{slope}$') is displayed as mean ± SD. P values are calculated using Mann-Whitney U test test compared to wildtype TRPC6. **Related to Figure 4.**

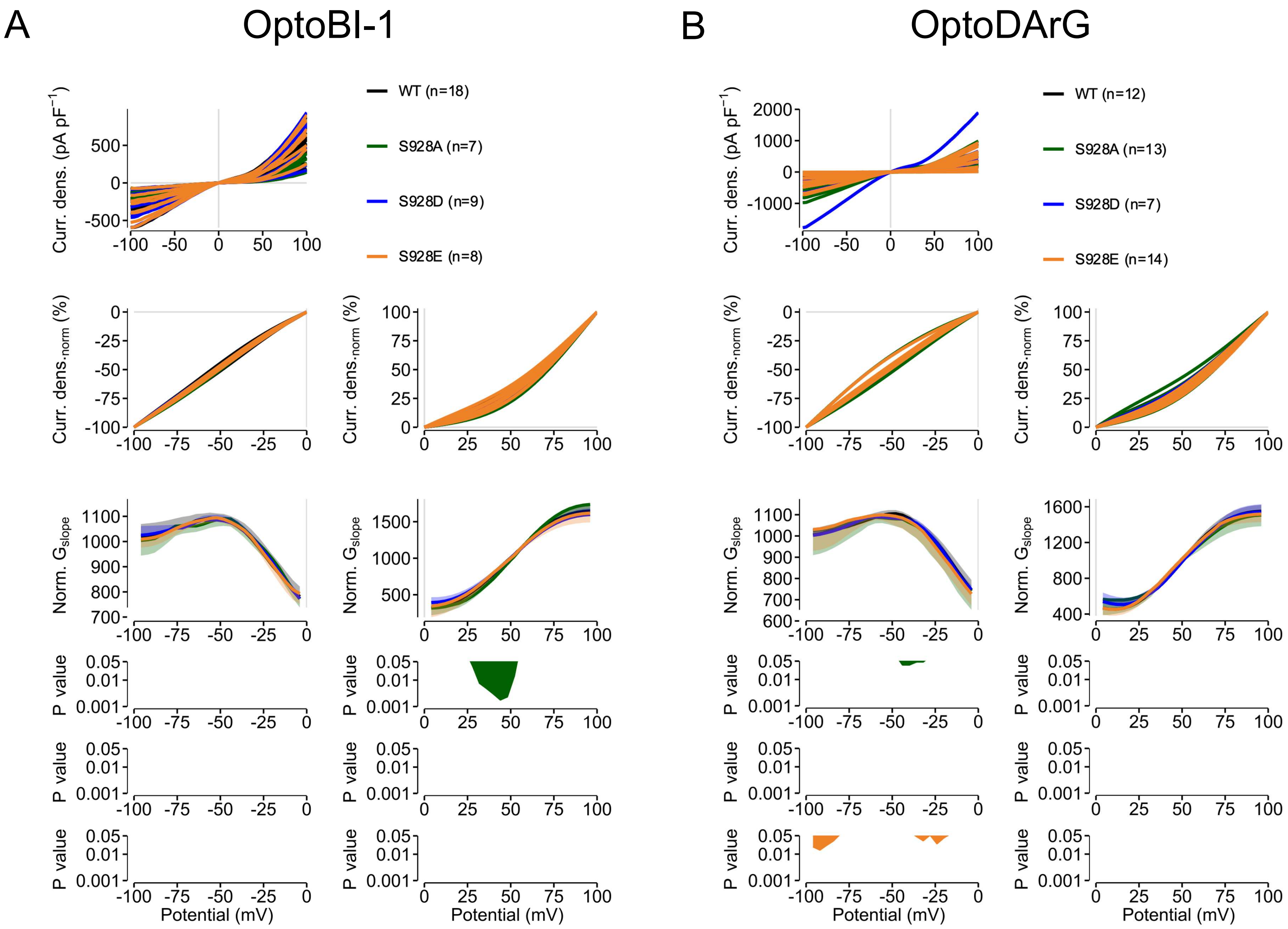


**Supplemental Figure 9: Normalized slope conductance of TRPC6 mutants with amino acid exchanges at S928.** Whole-cell measurements of TRPC6 and indicated TRPC6 mutants overexpressing HEK293T cells. (**A**, **B**) Current density-voltage relations ('Curr. dens.') induced by *cis*-OptoBI-1 (**A**) or *cis*-OptoDArG (**B**) are displayed above. The current density-voltage relations were separately smoothed and normalized ('Curr. dens$_{norm}$ (%)') at positive and negative potentials. The calculated normalized slope conductance (NSC) ('Norm. G$_{slope}$') is displayed as mean ± SD. P values are calculated using Mann-Whitney U test test compared to wildtype TRPC6. **Related to Figure 5.**

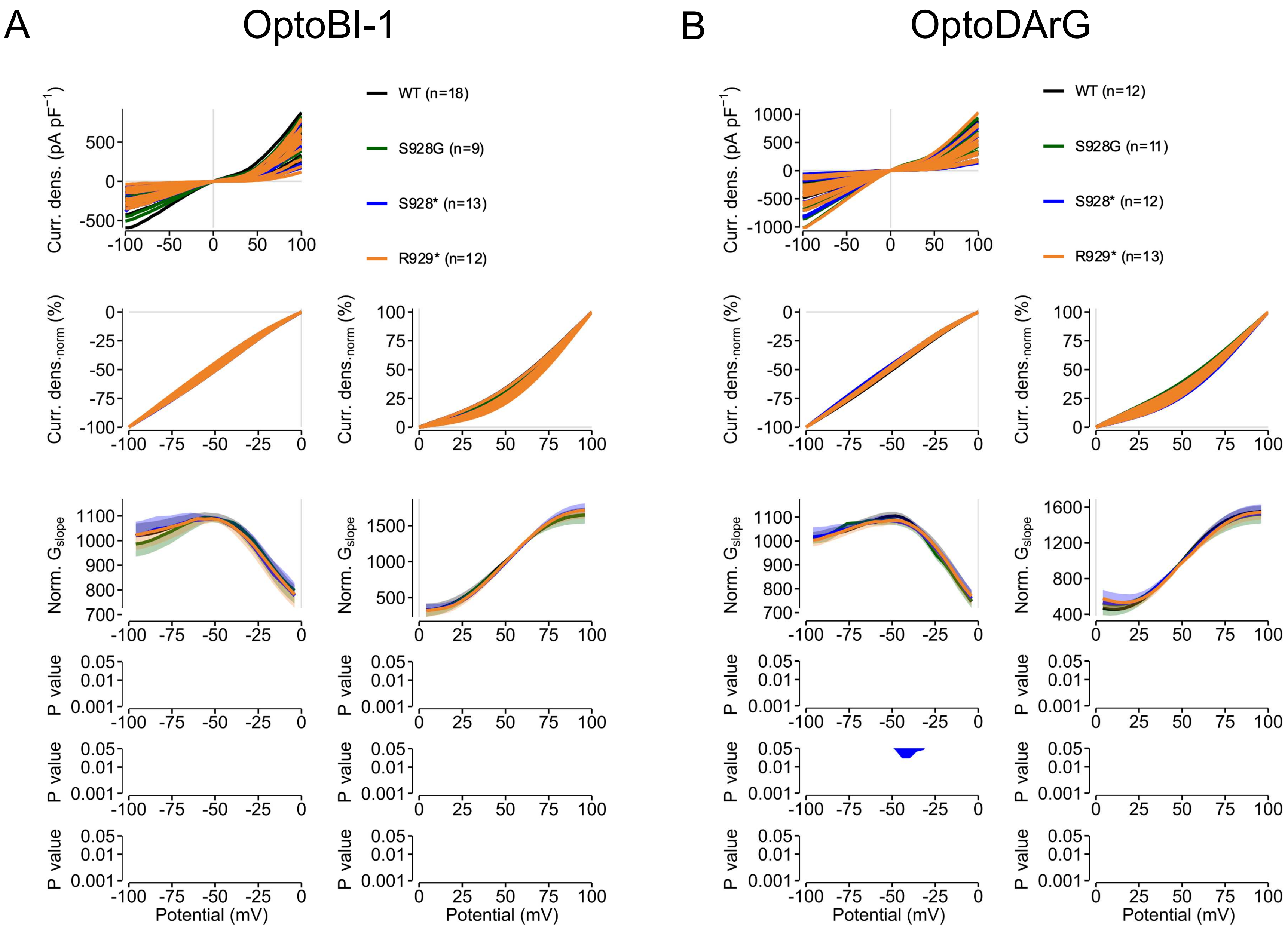


**Supplemental Figure 10: Normalized slope conductance of TRPC6 mutants with amino acid exchange at S928 or C-terminal truncation.** Whole-cell measurements of TRPC6 and indicated TRPC6 mutants overexpressing HEK293T cells. (**A**, **B**) Current density-voltage relations ('Curr. dens.') induced by *cis*-OptoBI-1 (**A**) or *cis*-OptoDArG (**B**) are displayed above. The current density-voltage relations were separately smoothed and normalized ('Curr. dens$_{norm}$ (%)') at positive and negative potentials. The calculated normalized slope conductance (NSC) ('Norm. $G_{slope}$') is displayed as mean ± SD. P values are calculated using Mann-Whitney U test test compared to wildtype TRPC6. **Related to Figure 6.**

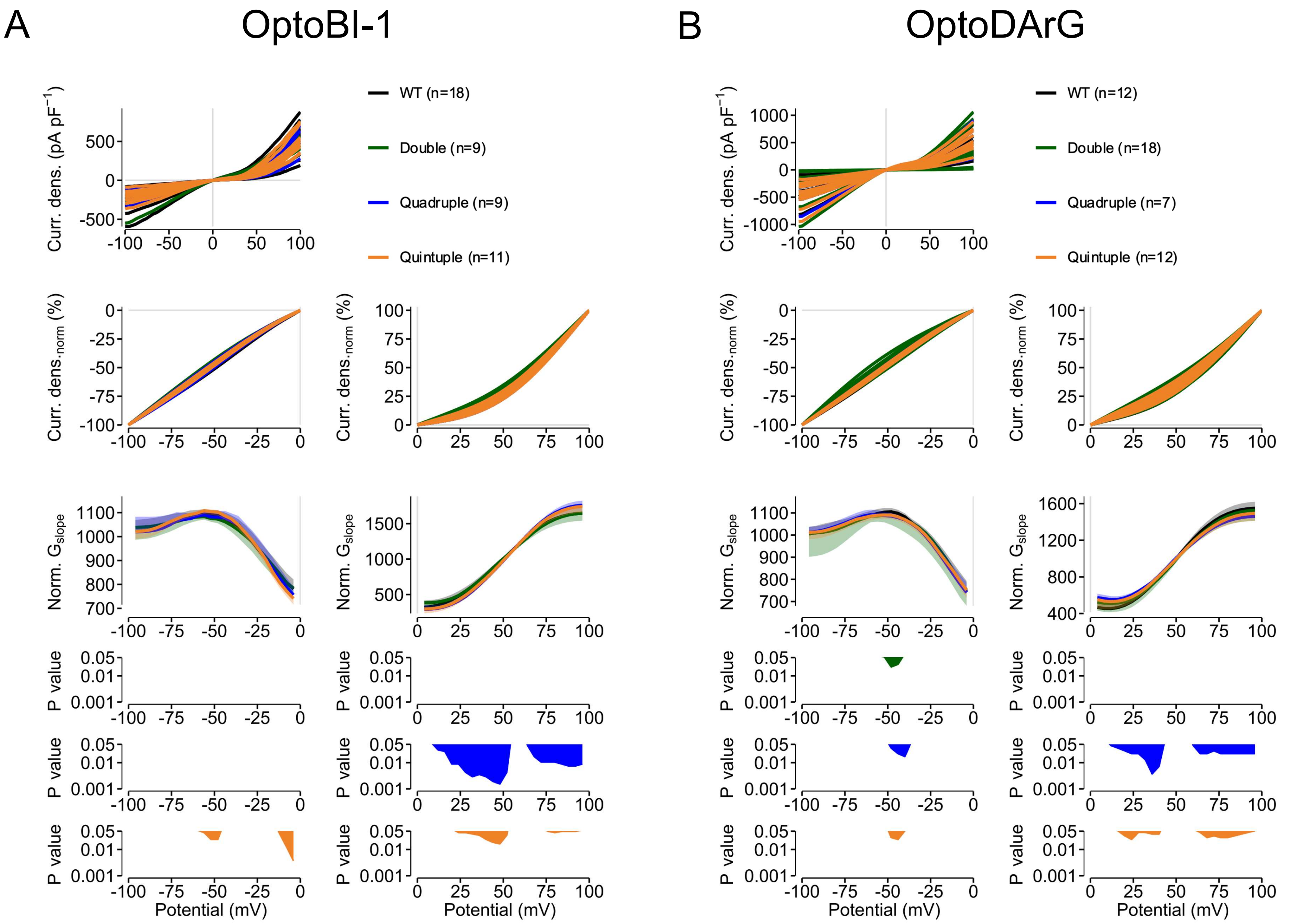


**Supplemental Figure 11: Normalized slope conductance of TRPC6 double, quadruple and quintuple mutants.** Whole-cell measurements of TRPC6 and indicated TRPC6 mutants overexpressing HEK293T cells. (**A**, **B**) Current density-voltage relations ('Curr. dens.') induced by *cis*-OptoBI-1 (**A**) or *cis*-OptoDArG (**B**) are displayed above. The current density-voltage relations were separately smoothed and normalized ('Curr. dens$_{norm}$ (%)') at positive and negative potentials. The calculated normalized slope conductance (NSC) ('Norm. G$_{slope}$') is displayed as mean ± SD. P values are calculated using Mann-Whitney U test test compared to wildtype TRPC6. **Related to Figure 7.**

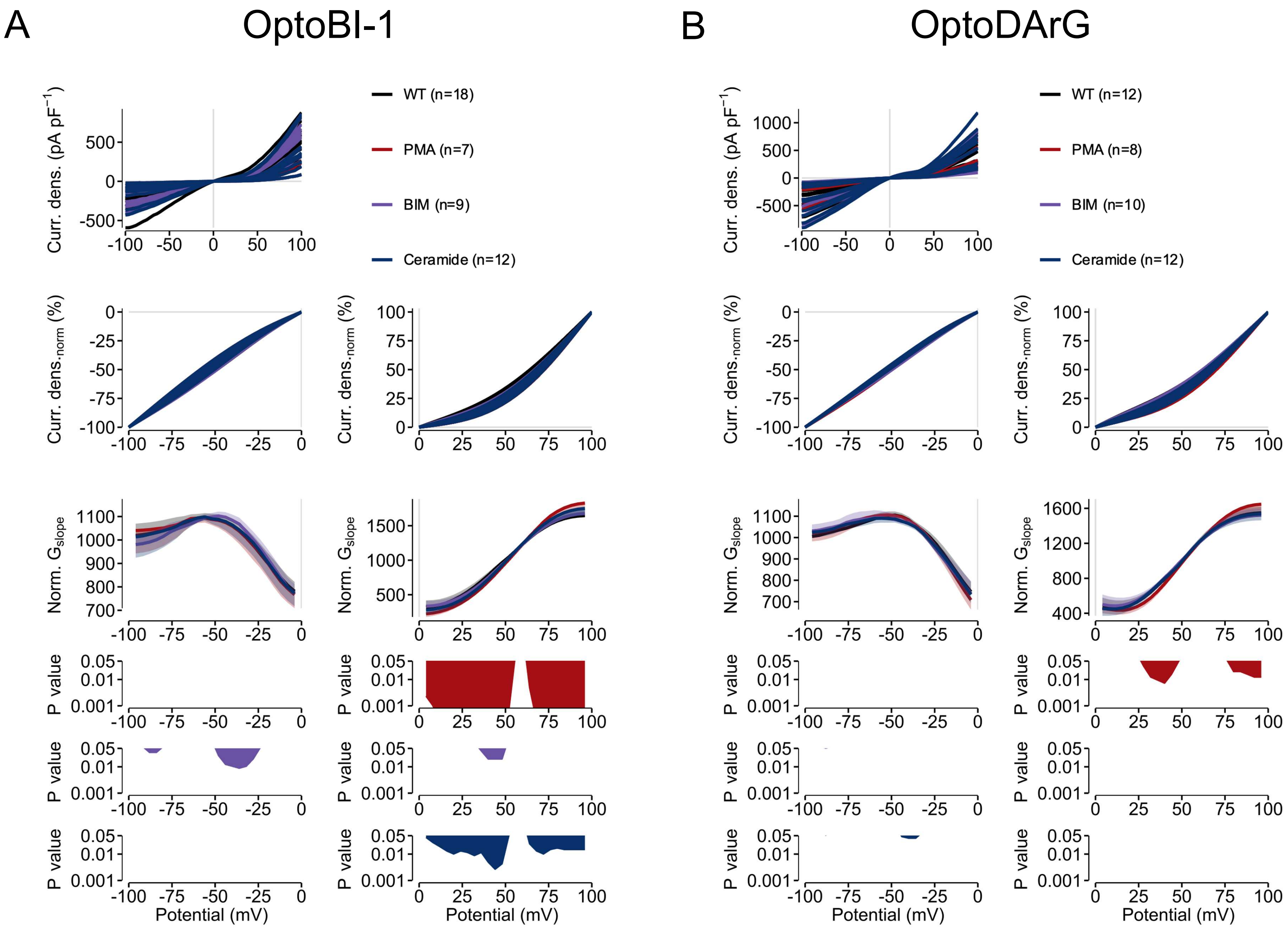


**Supplemental Figure 12: Normalized slope conductance of TRPC6 after PKC activation or inhibition.** Whole-cell measurements of TRPC6 overexpressing HEK293T cells after incubation with the PKC activator PMA (1 µM) or with the PKC inhibitors BIM I (1 µM) and ceramide (2 µM) for 20 minutes at room temperature. (**A**, **B**) Current density-voltage relations ('Curr. dens.') induced by *cis*-OptoBI-1 (**A**) or *cis*-OptoDArG (**B**) are displayed above. The current density-voltage relations were separately smoothed and normalized ('Curr. $dens_{norm}$ (%)') at positive and negative potentials. The calculated normalized slope conductance (NSC) ('Norm. $G_{slope}$') is displayed as mean ± SD. P values are calculated using Mann-Whitney U test. **Related to Figure 8.**

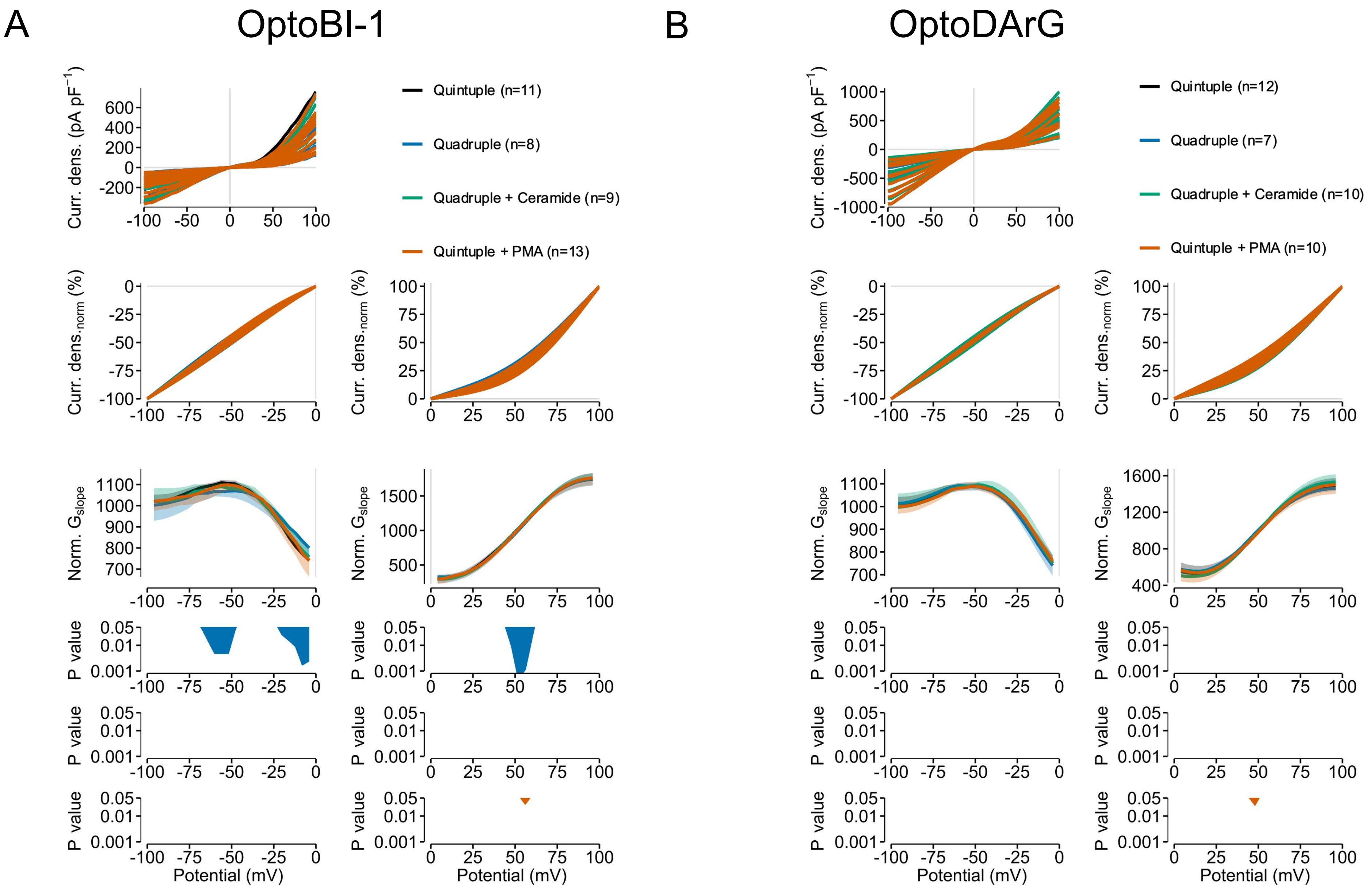


**Supplemental Figure 13: Normalized slope conductance of TRPC6 quadruple mutant after PKC inhibition and of quintuple mutant after PKC activation.** Whole-cell measurements of TRPC6 quadruple and quintuple mutants overexpressing HEK293T cells after incubation with the PKC activator PMA (1 µM) or with the PKC inhibitor ceramide (2 µM) for 20 minutes at room temperature. (**A**, **B**) Current density-voltage relations ('Curr. dens.') induced by *cis*-OptoBI-1 (**A**) or *cis*-OptoDArG (**B**) are displayed above. The current density-voltage relations were separately smoothed and normalized ('Curr. dens$_{norm}$ (%)') at positive and negative potentials. The calculated normalized slope conductance (NSC) ('Norm. G$_{slope}$') is displayed as mean ± SD. P values are calculated using Mann-Whitney U test compared to the quintuple mutant. **Related to Figure 9 and Figure 10.**
